# Integrated Transcriptomic and Metabolomic Analyses Reveal the Regulatory Networks of Cuticular Wax Fatty Acid Metabolism in Jujube (*Ziziphus jujuba* Mill. ‘Hupingzao’)

**DOI:** 10.3390/life16101685

**Published:** 2026-10-09

**Authors:** Na Li, Yuqin Song, Xinxin Feng, Aiqing Ji, Hongyan Han, Pengyu Ji, Liulin Li

**Affiliations:** 1 Department of Biological Science and Technology, Jinzhong University, Jinzhong 030609, China; 2 College of Horticulture, Shanxi Agricultural University, Taigu 030801, China

**Keywords:** very−long−chain fatty acids, β−Ketoacyl−CoA Synthase (KCS), wax metabolites, fruit development, gibberellin

## Abstract

The cuticular wax of ‘Hupingzao,’ an important economic crop in China, not only constitutes a key factor in determining the appearance quality and gloss of the fruit but also plays a vital role in regulating water evaporation and resisting biotic and abiotic stresses. In this study, ‘Hupingzao’ fruit produced during the white−ripe (WR) and full−red (FR) periods were used as study materials. The key genes and components of wax development were screened using a combination of transcriptomics and metabolomics. Enrichment analysis was performed on the differential genes and metabolites, and the fatty acid biosynthesis and elongation pathways were selected for further study. A series of key candidate genes in the de novo synthesis, elongation, and subsequent modification pathways of fatty acids were identified, such as *acetyl−CoA carboxylase* (*ACCase*), *fabF* (*β−Ketoacyl−ACP Synthase II*), and *β−ketoacyl−CoA synthase* (*KCS*). To further explore the mechanism of jujube wax biosynthesis, GC−MS (gas chromatography–mass spectrometry) was used to determine and analyze the content and composition of the wax after it was treated with gibberellin and paclobutrazol. The results showed that spraying the plants with GA_3_ significantly upregulated the expression of genes involved in fatty acid de novo synthesis and elongation such as *accC* (*Acetyl−CoA Carboxylase, Biotin Carboxylase Subunit*), *fabD* (*Malonyl−CoA:ACP Transacylase*), *fabG* (*β−Ketoacyl−ACP Reductase*), *fabI* (*Enoyl−ACP Reductase*), *ACSL* (*Long−chain Acyl−CoA Synthetase*), and *KCS* and increased the content of dodecanoic acid, behenic acid, lignoceric acid, and hexacosanoic acid. These results suggest that the GA signal may participate in the upregulation of fatty acid synthesis. In this study, we systematically constructed a molecular map of wax fatty acid metabolism during the fruit development stage of the ‘Hupingzao’ variety, providing an important theoretical basis and candidate genes for future directional regulation of wax components via molecular breeding or cultivation measures.

## 1. Introduction

The plant cuticle is a hydrophobic lipid structure covering the exterior of epidermal cells. It is primarily composed of cutin and cuticular wax [1]. Cuticular wax can be further categorized into intracuticular and epicuticular wax. Intracuticular wax is embedded within the dense, reticulated matrix of the cuticle, whereas epicuticular wax accumulates on the outermost surface and self−assembles into diverse crystalline structures (e.g., rods, columns, tubes, filaments, plates, and granules) [2]. Cuticular wax plays multiple physiological roles, including minimizing non−stomatal water loss, maintaining surface cleanliness and hydrophobicity, defending against pathogens and pests, mitigating damage from harmful radiation, and reflecting visible light [1].

Cuticular wax comprises very−long−chain fatty acids, aldehydes, ketones, esters, secondary alcohols, and alkanes, along with varying amounts of triterpenoids, sterols, and flavonoids [1,3]. There are significant differences in the morphological structures, quantity, and compositional profiles of cuticular waxes among different fruit species. Tomato cuticular wax, present on leaves, fruit, and anthers, primarily comprises cyclic compounds (e.g., terpenoids), alkanes, branched alkanes, and alkenes [4]. In contrast, the primary constituents of Chinese chive (*Allium tuberosum*) epicuticular wax are alkanes and ketones, whereas maize (*Zea mays*) and barley (*Hordeum vulgare*) leaf waxes lack these compound classes [5]. Apple (*Malus pumila*) wax is mainly composed of alkanes [6], litchi (*Litchi chinensis*) wax features fatty acids and primary alcohols [7], grape (*Vitis vinifera*) wax predominantly consists of oleanolic−acid−derived terpenoids [8], and Korla fragrant pear (*Pyrus sinkiangensis*) wax contains high proportions of alkanes and alkenes [9].

Cuticular wax content also varies considerably among cultivars. Vázquez et al. reported that 10,16−diOH C_16_:_0_ is the principal wax monomer in six mango (*Mangifera indica*) cultivars. Kent mangoes exhibited the highest concentration of this compound, followed by Ataulfo mangoes. Criollo and Tommy mangoes showed comparable proportions of 10,16−diOH C_16_:_0_ [10]. Similarly, Wu et al. identified 146 compounds in the fruit wax of 35 pear (*Pyrus*) cultivars, with long−chain alkanes constituting the major component. They noted sharp differences in the concentrations of alkanes, primary alcohols, fatty acids, esters, terpenoids, and aldehydes among five pear species. Furthermore, they found that the cuticular wax concentrations of hybrid pear cultivars were positively correlated with those of their parental lines [11]. Chai et al., examining the fruit wax of ten apple cultivars, confirmed that alkanes are the main compounds. Alkane content varied significantly among cultivars, with the highest and lowest levels detected in ‘Mishima Fuji’ and ‘Red Star’ apples, respectively [6].

The composition and quantity of cuticular wax also exhibit significant variation across different organs within the same plant species. Research on *Arabidopsis thaliana* has revealed distinct wax profiles among its organs: stems—alkanes (44%) > secondary alcohols (23%) > primary alcohols (12%) > ketones (9%) > aldehydes (7%) > esters (5%); rosette leaves—alkanes (73%) > aldehydes (14%) > primary alcohols (8%) > esters (4%); siliques—alkanes (64%) > secondary alcohols (11%) > primary alcohols (10%) > ketones (9%) > aldehydes (5%); flowers—alkanes (62%) > secondary alcohols (15%) > ketones (14%) > primary alcohols (5%); and seeds—alkanes (51%) > secondary alcohols (18%) > primary alcohols (13%) > ketones (9%) = aldehydes (9%) [12,13]. Similarly, studies on dandelion (*Taraxacum officinale*) indicate that there are organ−specific differences in wax coverage and composition. The wax mixtures on the beak and pappus exhibit similar compositional profiles, which are distinct from those found on the peduncle and petals. Notably, β−ketols were identified as components of the petal wax [14].

The synthesis and accumulation of cuticular waxes in fruits and other plant organs are coordinately regulated by environmental factors, including temperature [15], light [16], humidity [17], and hormones [18,19]. Notably, the composition, structure, and deposition of fruit cuticular wax undergo dynamic changes throughout fruit development, playing a decisive role in fruit morphogenesis, stress adaptation, and postharvest quality [20]. Studies on wolfberry (*Lycium barbarum*) have revealed period−specific wax profiles. The compositions of the cuticular wax of pericarps during the green fruit and color−break periods are identical, mainly consisting of alkanes, alcohols, and iodoalkanes (the last of which are rare components in plant waxes). In contrast, waxes from full−red fruit predominantly consist of alkanes, alcohols, and esters. Furthermore, comparative analysis has indicated that the fruit of the ‘Ningqi 5′ cultivar contains significantly higher proportions of alkanes in its cuticular wax relative to the ‘Ningqi 1′ cultivar. Given the established role of alkanes in enhancing fruit water retention capacity, ‘Ningqi 5′ is more suitable for drying [20]. Our previous research on ‘Hupingzao’ (*Ziziphus jujuba* Mill. ‘Hupingzao’) demonstrated distinct developmental shifts in wax composition. The highest relative proportions of fatty acids and alcohols were detected in the cuticular wax during the white−ripe period. Conversely, the highest proportion of fatty acids was observed during the coloring and full−red periods [21,22]. This trend is consistent with the wax characteristics of the ‘Summer Black’ grape (*Vitis vinifera* × *V. labrusca*) [23], Satsuma mandarin (*Citrus unshiu*), and Nanfeng tangerine (*Citrus reticulata* cv. Nanfeng) [24]. Importantly, fruits with high fatty acid wax content often exhibit decreased firmness, browning, flavor deterioration, and other undesirable postharvest phenomena [9].

‘Hupingzao’ (*Ziziphus jujuba* Mill. ‘Hupingzao’) belongs to the genus *Ziziphus* within the Rhamnaceae family. It is an ancient Chinese jujube cultivar of significant economic importance prized by consumers for its large fruit, vibrant color [25], sweet flavor [26,27,28], and rich nutritional profile [29,30]. Hailed as one of China’s “Ten Famous Jujube Cultivars,” ‘Hupingzao’ serves as a core pillar in regional agricultural economies and rural revitalization efforts. The composition of fruit surface wax, particularly its long−chain fatty acids (LCFAs), plays a vital role in mitigating fruit cracking and influences fruit storage, transportation, and processing quality. Therefore, investigating the biosynthetic mechanisms of cuticular wax in jujubes and understanding how wax composition is regulated are of paramount importance.

In this study, we utilized ‘Hupingzao’ fruit samples from the white−ripe and full−red periods as experimental materials. Integrated metabolomic and transcriptomic analyses were conducted to identify key metabolites and genes associated with cuticular wax biosynthesis in jujube. To further validate the identified key genes involved in wax synthesis, exogenous applications of GA_3_ and paclobutrazol were performed. Collectively, these findings provide a theoretical foundation for the subsequent regulation of cuticular wax synthesis. The results of this study will enrich our understanding of wax metabolism in jujube and offer a scientific basis for promoting high−quality development within the jujube industry.

## 2. Materials and Methods

### 2.1. Plant Material

Jujube (*Ziziphus jujuba* Mill. ‘Hupingzao’) fruits from the white−ripe (WR) and full−red (FR) periods were harvested from the Jujube Germplasm Resource Nursery, Pomology Institute, Shanxi Agricultural University, Shanxi, China. Fruit without disease or physical injuries was selected for sampling. All fruits were packed and delivered immediately to the laboratory at Shanxi Agricultural University.

Hormone treatments and sampling: Exogenous hormone treatments were applied during the young−fruit developmental period. A total of 9 uniform jujube trees, all more than 50 years old, were selected and randomly assigned to three groups—20 mg·L^−1^ GA_3_ (Solarbio, Beijing, China), 20 mg·L^−1^ paclobutrazol (PP_333_)(Solarbio, Beijing, China), and a distilled water control—with 3 trees per group. Whole jujube trees were foliar−sprayed with either 20 mg·L^−1^ of GA_3_ or 20 mg·L^−1^ of paclobutrazol (PP_333_), with 1 L of solution used per tree per application. Control trees were sprayed with an equivalent volume of distilled water. Applications were repeated in 2−day intervals for a total of three treatments. Fruit sampling was conducted in the white−ripe and the full−red periods. For each treatment and each sampling period, 9 fruits were collected from 3 trees, yielding 3 biological replicates. No technical replicates were employed. The resulting samples were designated as follows: WR−CK, white−ripe period control; FR−CK, full−red period control; FR−GA_3_, full−red period, GA_3_−treated; and FR−PP_333_, full−red period, paclobutrazol−treated.

### 2.2. Transcriptomic Analysis

For each developmental period (the white−ripe and full−red periods), three biological replicates were collected. Each biological replicate consisted of pericarp pooled from 3 fruits. The total RNA from each sample was extracted from the pericarp via the cetrimonium bromide (CTAB) method. Qualified RNA samples were selected for high−throughput sequencing. Transcriptome sequencing was performed by Shanghai Lianchuan Biotechnology Co., Ltd. After data quality control, clean reads were obtained. The sequencing reads were aligned to the jujube reference genome (BioProject accession: PRJNA1014986) using HISAT2 (Hierarchical Indexing for Spliced Alignment Tool 2). We used FeatureCounts to quantify raw integer read counts mapped to the reference genome after filtering out reads with a mapping quality score below 10. These raw counts were used as input for DESeq2 to identify differentially expressed genes (DEGs), with significance defined as an adjusted *p*−value < 0.05 and |log_2_FC| ≥ 1 (i.e., at least a two−fold change). FPKM (fragments per kilobase of exon model per million mapped fragments) values were calculated separately based on gene length and used only for descriptive visualization of gene expression levels. The DEGs were functionally annotated using the Gene Ontology (GO) database, and metabolic pathway enrichment analysis was performed using the Kyoto Encyclopedia of Genes and Genomes (KEGG) database, with a significance threshold of *p* < 0.05.

### 2.3. Metabolomics Analysis

For each developmental period (the white−ripe and full−red periods), three biological replicates were collected. Each biological replicate consisted of pericarp pooled from 3 fruits. Following fruit collection, the peel was excised. We used a freeze−drying machine to vacuum freeze−dry the skin before grinding it into powder. A total of 50 mg of the lyophilized powder was dissolved in 1.2 mL of 70% (*v*/*v*) methanol extraction solvent. The mixture was vortexed for 30 s every 30 min for a total of six cycles. After centrifugation at 12,000 rpm for 3 min, the supernatant was collected, filtered through a 0.22 µm microporous membrane, and stored for subsequent metabolomic analysis. Non−targeted metabolite profiling was performed using the ACQUITY UPLC system (Waters, Milford, MA, USA). A high−resolution tandem mass spectrometer TripleTOF 6600 (SCIEX, Framingham, MA, USA) was used to detect metabolites eluted form the column. Raw mass−spectrometry data files were converted into mzXML format using MSConvert software (ProteoWizard). Peak extraction and quality control were performed with XCMS software (https://xcmsonline.scripps.edu/landing_page.php?pgcontent=mainPage, accessed on 20 April 2025). The CAMERA tool was used for adduct ion annotation of the extracted substances (https://github.com/sneumann/CAMERA, accessed on 20 April 2025). MetaX software was applied for primary identification (https://github.com/wenbostar/metaX, accessed on 20 April 2025). The identification processes included both MS1−based annotation and MS2 spectral matching against an in−house standard library. entification, the adducts in positive mode included [2M+H]+, [M+H]+, [M+K]+, [M+Na]+, and [M+NH_4_]+, while in negative mode, [M−H]−, [M+Cl]−, [2M−H]−, [M+CHO_2_]−, and [M+C_2_H_3_O_2_]− were considered. The MS1 mass tolerance was set to 10 ppm. The databases used for matching included an in−house database and common open−source databases (e.g., HMDB, MassBank, and LipidMaps, depending on the pro−ject). Candidate metabolites from MS1 were further validated by MS2 fragmentation. The mass tolerances for both MS1 and MS2 were set to 20 ppm, and the identification score cut−off was set to 70%. The MS/MS fragmentation spectra of the samples were matched against the in−house database and open−source databases. The LC−MS conditions and data−processing technique employed were based on the study by Lang et al. [31]. Candidate identified substances were annotated using databases such as HMDB (Human Metabolome Database) and KEGG. MetaX software was used to quantify and screen differential metabolites, with *p* < 0.05 and |log_2_FC| ≥ 1 for significant differential metabolites set as criteria.

Integrated transcriptomic–metabolomic analysis: To explore potential regulatory relationships, integrative analysis was conducted on the transcriptomic and metabolomic datasets. Differentially expressed genes (DEGs) and differentially abundant metabolites (DAMs) were independently subjected to KEGG pathway enrichment analysis. Significantly co−enriched pathways (*p* < 0.05) were identified as potential points of biological interaction between gene expression and metabolite abundance.

### 2.4. Qualitative and Quantitative Analysis of Cuticular Wax Components

For GC−MS analysis of wax components, three biological replicates were analyzed for each stage/treatment. Each biological replicate consisted of 3 fruits. Each sample was analyzed once (there were no technical replicates). We referred to Li et al. regarding the methods used for the qualitative and quantitative analysis of waxy components [22]. 

Determination of wax and cuticle mass: Fruits were rinsed with distilled water and peeled. The CM was isolated by incubating the peel in 50 mM citric acid buffer (pH 3.8) containing pectinase (0.2 g/L, Solarbio, Beijing, China), cellulase (0.6 g/L, Solarbio, Beijing, China), and NaN_3_ (0.13 g/L, Jianglai, Shanghai, China) at room temperature. The solution was replaced three times until the CM separated completely. The CM was washed with distilled water and then dried at 30 °C until it reached a constant weight. A 0.10 g subsample of dried CM was extracted with 5 mL of n−hexane (Solarbio, Beijing, China) at 30 °C for 6 h. The extract was filtered through a 0.45 µm organic membrane and dried under nitrogen at 30 °C.

Qualitative and quantitative analysis of CM composition: The dried extract was derivatized with 200 µL of pyridine (Solarbio, Beijing, China) at 50 °C for 30 min, followed by BSTFA (SigmaAldrich, China) at 60 °C for 40 min. After 2 μg of n−tetracosane (tetracosane) was added as the internal standard, the mixture was evaporated under nitrogen at 30 °C. An aliquot (1 µL) was analyzed using a gas chromatograph–mass spectrometer (DSQII; Thermo Fisher Scientific, Waltham, MA, USA) equipped with a TR−1MS column (30 m × 0.25 mm i.d., 0.25 µm, 100% dimethyl polysiloxane). MS was conducted in positive EI mode at 70 eV with an m/z range of 50–650. The injector, ion source, and transfer line temperatures were 250, 300, and 250 °C, respectively. Helium was used as the carrier gas at 1.2 mL/min without a split. The temperature program was as follows: 70 °C for 2 min, 10 °C/min up to 200 °C (2 min), 2 °C/min up to 285 °C, 1 °C/min up to 290 °C, 2 °C/min up to 300 °C (10 min), and 3 °C/min up to 320 °C (20 min). GC−MS analysis was performed within 10 h.

### 2.5. Promoter Elements Analysis

The 2.0 kb DNA length upstream of the coding sequence of candidate genes was used to identify promoter cis−elements. PlantCARE software (http://bioinformatics.psb.ugent.be/webtools/plantcare/html/, accessed on 20 April 2025) was used to search for cis−acting regulatory elements.

### 2.6. Quantitative Real−Time PCR Validation of Wax Metabolism Gene Expression Levels

Total RNA extraction and gene expression detection were performed as described in Li et al.’s work [22]. The gene symbols used in this study correspond to the nomenclature of their closest homologs in the plastid/E. coli fatty acid synthesis pathway and were used solely for functional annotation. The corresponding jujube gene IDs, ortholog identity, accession numbers, and primers used in this study are provided in Supplementary Material Table S1. The jujube *Histon−H* (*HIS*) gene (GenBank accession No. EU916201) was used as an internal control to normalize all mRNA expression levels. The 2^−△△Cq^ method was used to calculate the relative amount of template present in each PCR amplification mixture.

### 2.7. Data Analysis

All experiments were performed with at least three biological replicates. Data are presented as means ± standard deviations (SDs). One−way analysis of variance (ANOVA), followed by Duncan’s multiple−range test, was performed, with *p* < 0.05 considered statistically significant. The different lowercase letters (a, b, and c) in the figures indicate significant differences among groups. All statistical analyses were performed using SAS System 8.6 (SAS Institute, Cary, NC, USA). The figures were constructed using Microsoft Excel 2021, Microsoft PowerPoint 2021, and OriginPro 2024 (Microcal Software Inc., Northampton, MA, USA).

## 3. Results

### 3.1. Transcriptome Sequencing and Differential Gene Analysis

**Figure 1:**
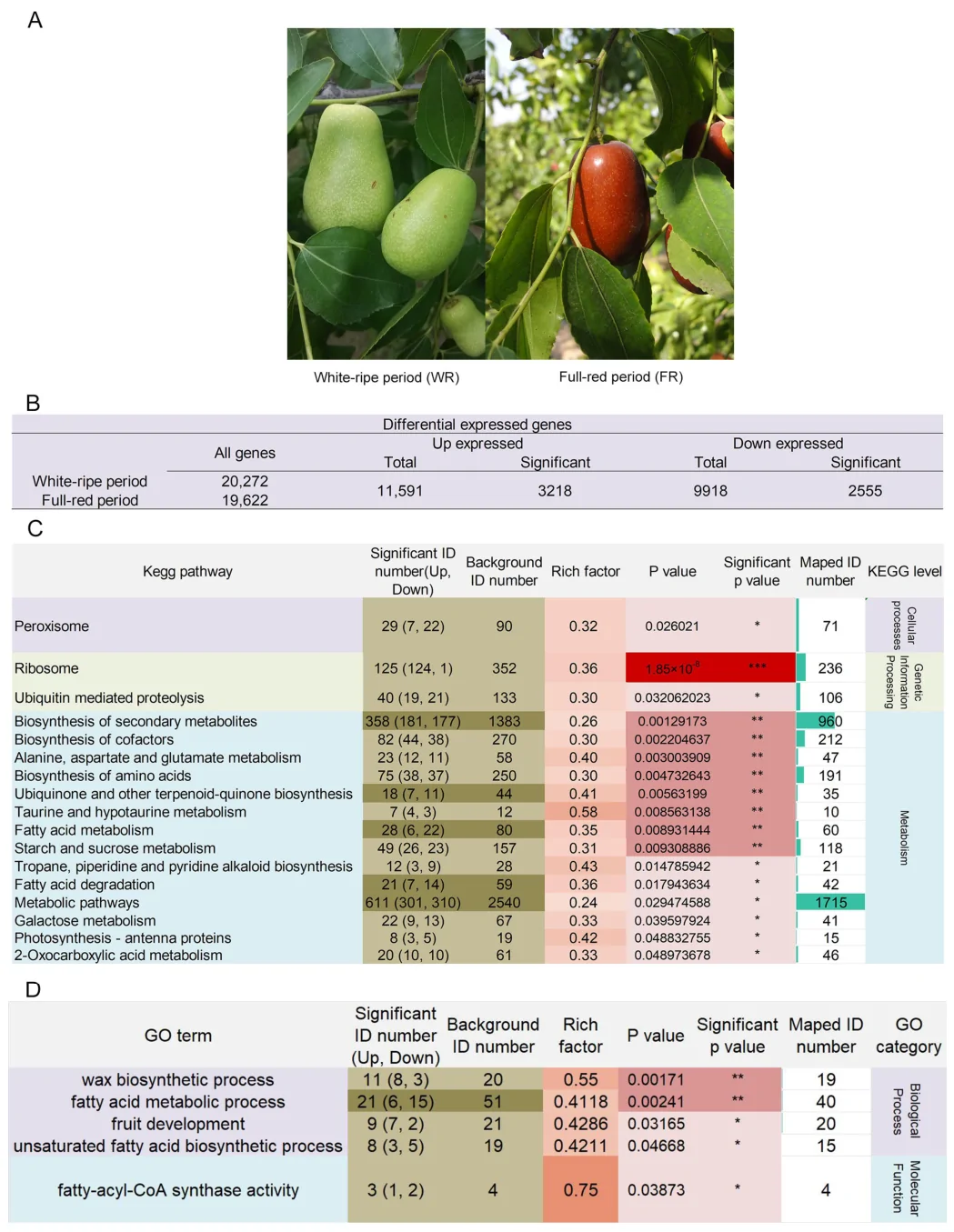
Analysis of differentially expressed genes (DEGs) during ‘Hupingzao’ development. (**A**): Photos of ‘Hupingzao’ fruit during the whiteripe (WR) and full−red (FR) periods. (**B**): Statistics regarding the differentially expressed genes between the white−ripe and full−red periods. (**C**): Enrichment—Kyoto Encyclopedia of Genes and Genomes (KEGG). (**D**): Enrichment—Gene Ontology (GO). Note: Significance levels are indicated by ***, **, and * for *p*−value ranges of [0.0001, 0.001), [0.001, 0.01), and [0.01, 0.05), respectively.

Inter−development period differences in gene expression patterns were detected via RNA−Seq, and transcriptome sequencing was used to investigate the gene expression patterns of ‘Hupingzao’ during the white−ripe and full−red periods (Figure 1A). According to the transcriptome analysis, 33,738,016 and 54,472,066 raw reads were obtained from the ‘Hupingzao’ fruit samples during the two periods, and 26,342,474 and 52,005,642 clean reads were obtained after filtering. The content of Q20 was 99.22% and 99.30%, and the content of Q30 was 96.23% and 96.02%, respectively. Both GC contents were 45% (Supplementary Materials Table S2). In total, 20,272 and 19,622 genes were expressed in ‘Hupingzao’ during the white−ripe and full−red periods, respectively (expression threshold = 1) (Figure 1B). Overall, 11,591 and 9918 genes were upregulated and downregulated, respectively. Among them, 3218 genes and 2555 genes were differentially expressed genes (DEGs) (as defined in Section 2.2).

Kyoto Encyclopedia of Genes and Genomes (KEGG) pathway analysis: All annotated genes were mapped to KEGG pathways for functional classification. DEGs were then subjected to KEGG enrichment analysis, with the complete set of annotated genes used as the background. The hypergeometric test was used, and pathways with *p* < 0.05 were considered significantly enriched (Figure 1C). KEGG analysis classified DEGs under cellular processes, genetic information processing, and metabolism. Cellular processes encompassed a total of 71 genes, including 29 differentially expressed genes (7 upregulated and 22 downregulated). Genetic information processing was annotated by two pathways, ribosome− and ubiquitin−mediated proteolysis. Among them, 236 and 106 genes were compared, with 125 and 10 genes showing differential expression, respectively. A total of 14 pathways were annotated in the metabolism classification. The three pathways this study focuses on—biosynthesis of secondary metabolites, fatty acid metabolism, and fatty acid degradation—were compared to 960, 60, and 42 genes, respectively, with 358, 28, and 21 genes showing differential expression.

Gene Ontology (GO) analysis: All genes were analyzed using GO enrichment, and the GO pathways for which there was a significant *p* value < 0.05 are shown in Supplementary Materials Table S3. The results were divided into three categories: biological process, cellular component, and molecular function. In total, 139, 43, and 89 pathways were annotated to each term, respectively. The obsolete oxidation–reduction process had the most annotated genes (226 genes) under biological process; cytoplasm had the most annotated genes (844 genes) under cellular component; and metal ion binding had the most annotated genes (279 genes) under molecular function (Supplementary Materials Table S3). The GO terms with the strongest enrichment significance for biological process, cellular component, and molecular function were translation, cytosolic large ribosomal subunit, and structural constituent of ribosome. The *p*−values were 1.39 × 10^−11^, 8.04 × 10^−18^, and 3.71 × 10^−18^, respectively. Among all the GO pathways with *p* < 0.05, we identified five GO terms that were related to the wax metabolism of interest in this study (Figure 1D). Wax biosynthetic processes, fatty acid metabolic processes, fruit development, and unsaturated fatty acid biosynthetic processes are classified under the biological process category, while fatty−acyl−CoA synthase activity is classified under molecular function. Wax biosynthetic processes, fatty acid metabolic processes, fruit development, unsaturated fatty acid biosynthetic processes, and fatty−acyl−CoA synthase activity were enriched in 11, 21, 9, 8, and 3 genes, respectively, with enrichment factors of 0.55, 0.41, 0.43, 0.42, and 0.75, respectively.

### 3.2. Differential Metabolite Screening and Enrichment Analysis

**Figure 2:**
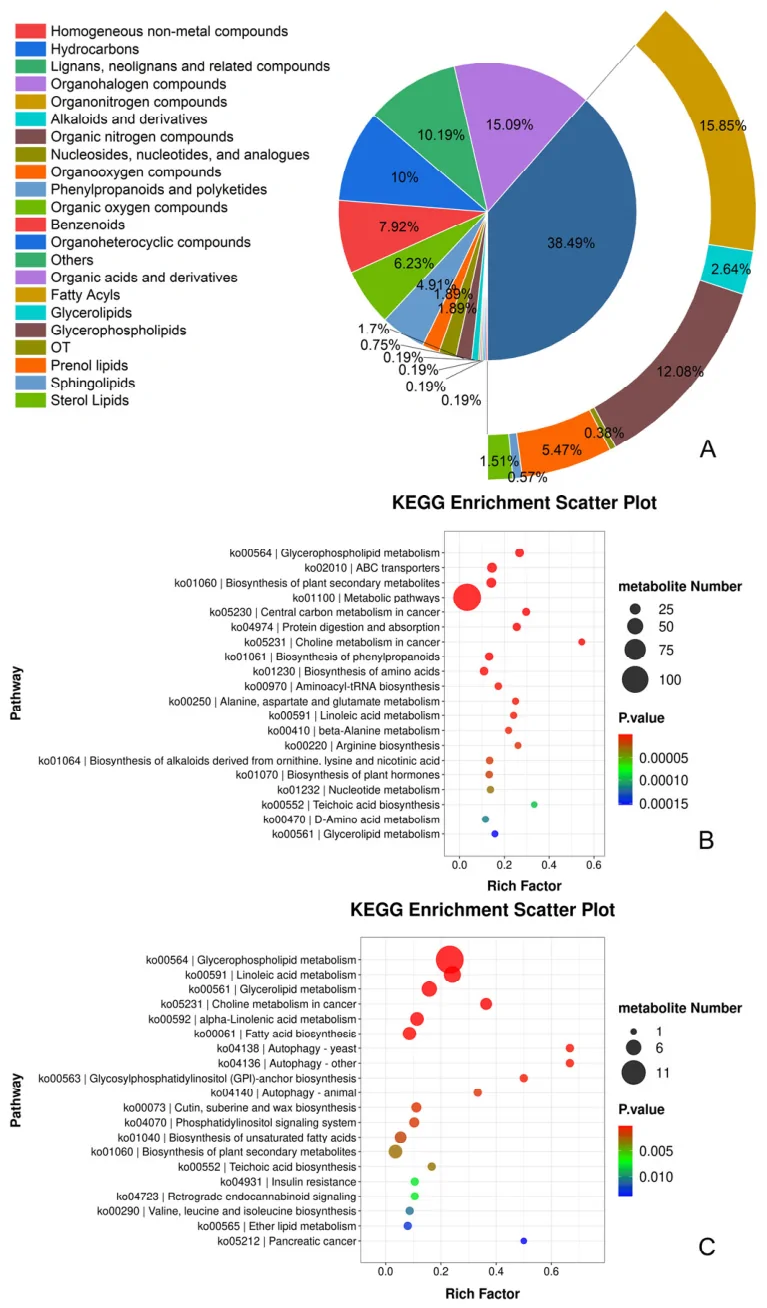
Metabolite statistics. (**A**): Metabolite−type statistics. (**B**): Top 20 KEGG pathways enriched among the annotated metabolites. (**C**): Enrichment analysis of 117 metabolites annotated by KEGG in the ‘lipid and lipid molecular metabolites’ category.

Significantly different metabolites: We identified total of 530 metabolites in the ‘Hupingzao’ fruits in the white−ripe (WR) and full−red (FR) periods. These metabolites could be classified into 16 categories. Among them, ‘Lipids and lipid−like molecules’ had the most metabolites (204), accounting for 38.49% of all types of metabolites. ‘Organic acids and derivatives’ ranked second, accounting for 15.09% of the total. Metabolites classified as ‘Organoheterocyclic compounds’, ‘Benzenoids’, ‘Organic oxygen compounds’, ‘Phenylpropanoids and polyketides’, ‘Organooxygen compounds,’ and ‘Nucleosides, nucleotides and analogues’ were also detected (Figure 2A). The classification of ‘Lipids and lipid like molecules’ was further divided into seven subcategories: ‘Fatty Acyls’, ‘Glycerolipids’, ‘Glycerophospholipids’, ‘Others’, ‘Prenol Lipids’, ‘Sphingolipids’, and ‘Sterol Lipids’. The seven subcategories contain 84, 14, 64, 2, 29, 3, and 8 metabolites, respectively, accounting for 15.85%, 2.64%, 12.08%, 0.38%, 5.47%, 0.57%, and 1.51% of all metabolites.

Among the 530 metabolites, 257 could be annotated to the KEGG pathway. Enrichment analysis was performed on 257 metabolites (Supplementary Materials Table S4). The first 20 KEGG pathways ranked according to enrichment significance from small to large are shown in Figure 2B. The enrichment differences were extremely significant in 53 pathways and significantly different in 37 pathways (Supplementary Materials Table S4). The three pathways with the most IDs were ‘Metabolic pathways (106),’ ‘Biosynthesis of secondary metabolites (55),’ and ‘Microbial metabolism in diverse environments (25)’. Pathways enriched in the secondary classifications of ‘Biosynthesis of other secondary metabolites’, ‘Amino acid metabolism’, and ‘Lipid metabolism’ accounted for the greatest quantities, with 21, 14, and 14 pathways, respectively.

We performed enrichment analysis on 117 metabolites that could be annotated via KEGG from the category ‘Lipids and lipid like molecules metabolites’. On the basis of the significance of enrichment, scatter plots were created for the top 20 KEGGs from small to large (Figure 2C). There were two pathways related to wax metabolism: ‘Fatty acid biosynthesis’ and ‘Cutin, suberine and wax biosynthesis’. The *p*−values were 4.45 × 10^−5^ and 8.11 × 10^−4^, respectively. The enrichment factors were 0.09 and 0.11, respectively. The two pathways ‘Fatty acid biosynthesis’ and ‘Fatty acid elongation’ were further analyzed.

### 3.3. Analysis of the Fatty Acid Biosynthesis Pathway

**Figure 3:**
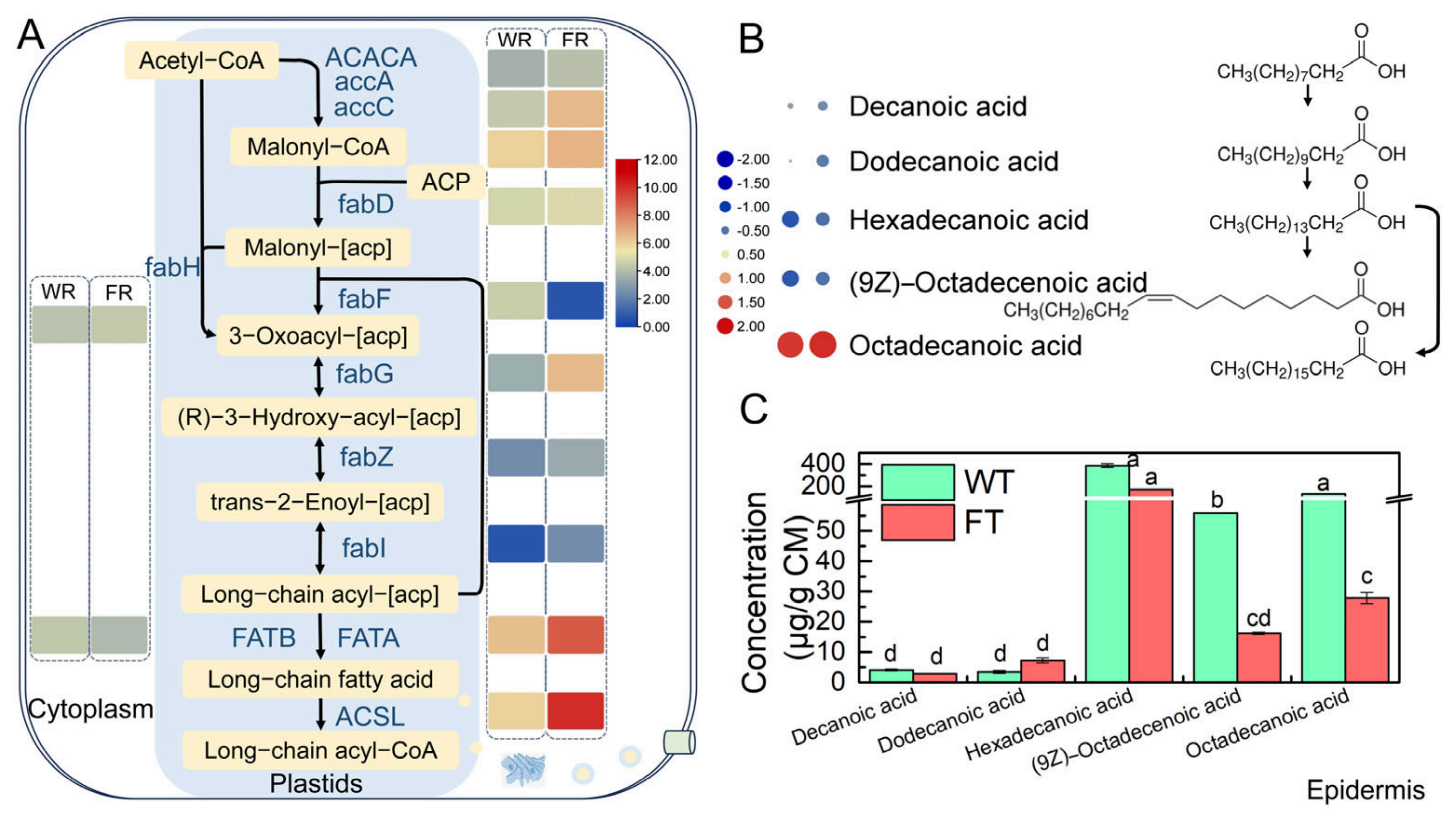
Pathway analysis correlating the transcriptome and metabolome in the fatty acid biosynthesis of jujube fruit during the white−ripe and full−red periods. (**A**): RNA−Seq outcomes of the pathway related genes. Red indicates high expression (high FPKM values), and blue indicates low expression (low FPKM values). (**B**): The fatty acid metabolites identified through metabolomic analysis. (**C**): Compositional analysis of epicuticular waxes. CM: cuticular membrane (defined in the Materials and Methods (Section 2.4)). Data are presented as means ± SDs (*n* = 3 biological replicates). Different lowercase letters indicate significant differences among treatments as determined via one−way ANOVA followed by Duncan’s multiple−range test (*p* < 0.05).

Fatty acids are biosynthesized in the cytoplasm. In this study, the synthesized long−chain acyl−CoAs were subsequently transferred to the endoplasmic reticulum for further elongation until the formation of long−chain fatty acids and their derivatives, which were ultimately exported extracellularly. As illustrated in Figure 3A, fatty acid synthesis initiates with acetyl−CoA as the substrate and proceeds through multiple catalytic cycles to generate C18 fatty acids and C18 acyl−CoAs. Notably, certain steps in this process involve multiple enzymes acting on the same reaction. With the exception of *fabF*, the FPKM values of 11 other genes (*accA*, *fabG*, *fabZ* (*β−Hydroxyacyl−ACP Dehydratase*), *fabI*, *FATA* (*Acyl−ACP Thioesterase A*), and *ACSL*) were significantly higher during the full−red period relative to the white−ripe period. Conversely, the FPKM value of *fabF* was markedly elevated in white−ripe fruits relative to full−red fruits.

Figure 3B presents the fatty acid metabolites identified through metabolomic analysis, including decanoic acid (C10H20O2), dodecanoic acid (C12H24O2), hexadecanoic acid (C16H32O2), (9Z)−octadecenoic acid (C18H34O2), and octadecanoic acid (C18H36O2). (These metabolites were putatively annotated in the wax fraction based on MS1/MS2 spectral matching. Future studies will validate these assignments and revise the biological interpretation if necessary.) (9Z)−octadecenoic acid is an unsaturated long−chain fatty acid, while octadecanoic acid exhibited substantially higher abundance relative to the other four fatty acids. Notably, all five fatty acids demonstrated greater accumulation during the white−ripe period than in the full−red period.

The compositional analysis of epicuticular waxes (Figure 3C) revealed that white−ripe fruits had significantly higher (9Z)−octadecenoic acid and octadecanoic acid content than full−red fruits. Interestingly, hexadecanoic acid predominated in fruit surface waxes, showing a distribution pattern inconsistent with the metabolomic results. Supplementary Materials Table S5 details the proportional composition of wax components in ‘Hupingzao’ jujube fruits. Fatty acids constituted 57.82% of the total wax components in white−ripe fruits, decreasing to 30.27% at maturity, while primary alcohols maintained stable proportions (27.73% vs. 26.70%). The full−red fruit exhibited increased ester content (21.80%).

These findings suggest that C16 fatty acids are the principal components of epicuticular waxes. White−ripe fruit epidermis appears to utilize abundant C18 fatty acids as substrates for synthesizing other wax constituents, whereas full−red fruits exhibit reduced C18 fatty acid content alongside increased accumulation of alternative wax components.

### 3.4. Analysis of the Long−Chain−Fatty−Acid Extension Pathway

**Figure 4:**
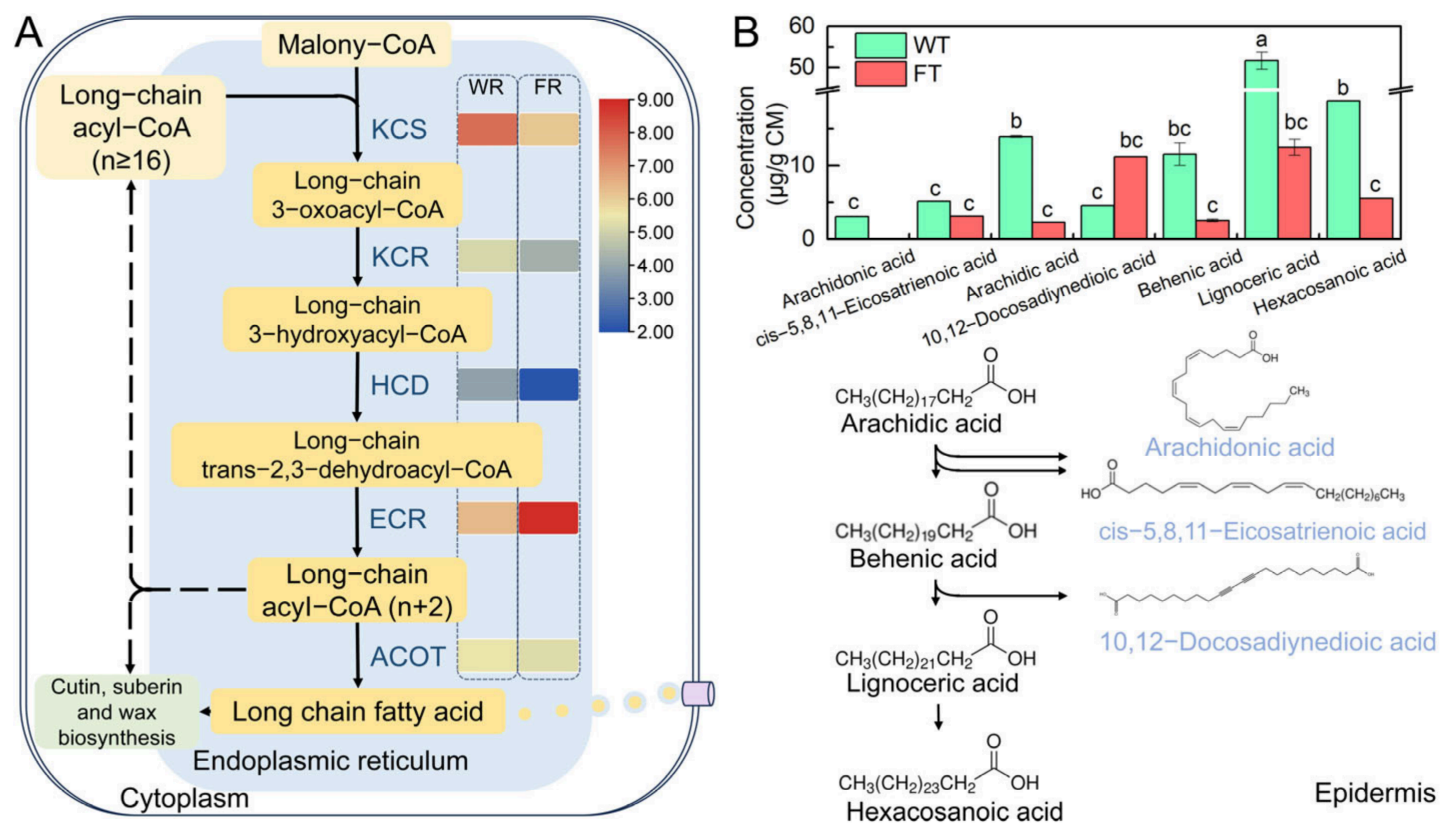
Pathway analysis correlating the transcriptome and metabolome in the fatty acid elongation of jujubes at the white−ripe and full−red stages. (**A**): RNA−Seq outcomes of the pathway−related genes. (**B**): Compositional analysis of epicuticular waxes. CM: cuticular membrane. Data are presented as means ± SDs (*n* = 3 biological replicates). Different lowercase letters indicate significant differences among treatments determined via one−way ANOVA followed by Duncan’s multiple−range test (*p* < 0.05).

Figure 4A illustrates the relative expression levels of genes involved in the long−chain−fatty−acid elongation pathway. The FPKM values of *KCS*, *KCR* (*β−ketoacyl−CoA reductase*), *HCD* (*β−hydroxyacyl−CoA dehydratase*), and *ACOT* (*Acyl−CoA Thioesterase*) were higher in white−ripe fruits than in full−red fruits, with *KCS* and *HCD* exhibiting 3.02−fold and 4.18−fold differences, respectively. In contrast, the FPKM value of *ECR* (*enoyl−CoA reductase*) was lower in white−ripe fruits (corresponding to a 6−fold reduction relative to the full−red fruit). Metabolomic analysis failed to detect metabolites associated with the long−chain−fatty−acid elongation pathway.

Figure 4B displays the contents of five fatty acids in epicuticular wax components. Seven long−chain−fatty−acid products were identified extracellularly. Arachidonic acid, cis−5,8,11−eicosatrienoic acid, arachidic acid, behenic acid, lignoceric acid, and hexacosanoic acid (These metabolites were putatively annotated in the wax fraction based on MS1/MS2 spectral matching. Future studies will validate these assignments and revise the biological interpretation if necessary.) were more abundant in white−ripe fruits than in full−red ones, with arachidic acid, lignoceric acid, and hexacosanoic acid showing statistically significant differences (*p* < 0.05). Conversely, 10,12−docosadiynedioic acid exhibited higher abundance in full−red fruits, though this difference was not significant.

### 3.5. The Related Cis Elements of the Long−Chain−Fatty−Acid Metabolism Genes

**Figure 5:**
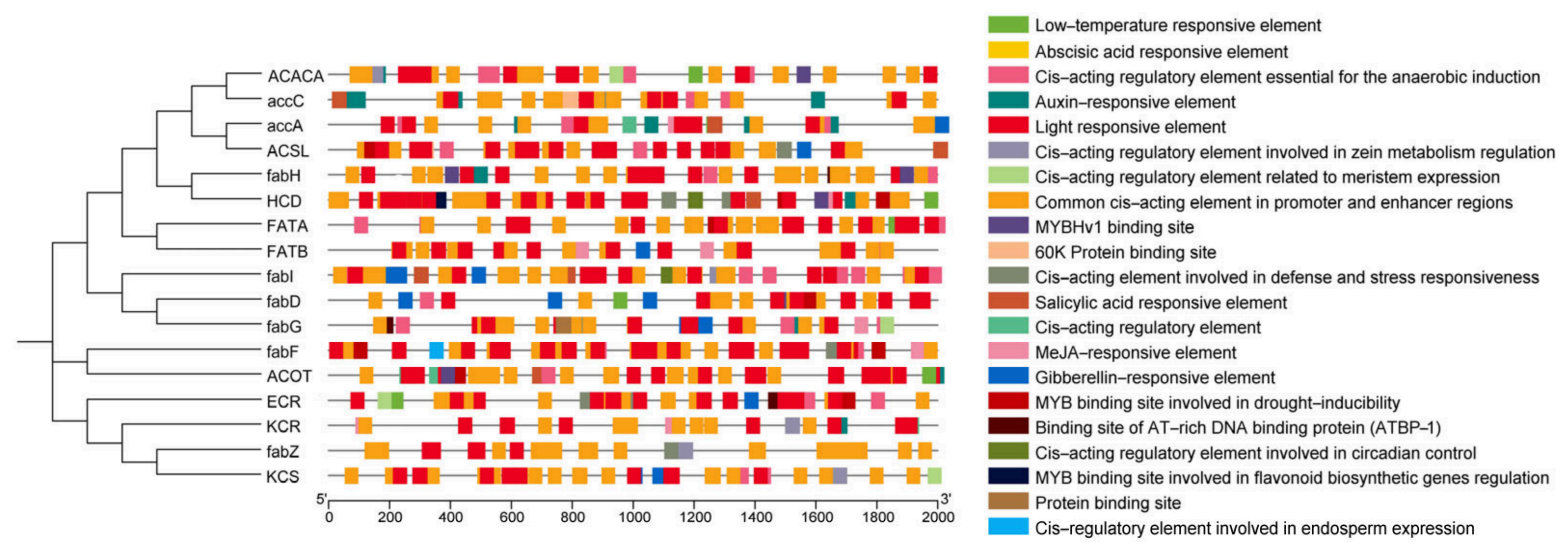
Related cis−elements of the long−chain−fatty−acid metabolism genes.

Phylogenetic analysis of fatty−acid−metabolism−related genes: The protein sequences encoded by the 17 jujube genes associated with long−chain−fatty−acid metabolism were aligned using MEGA 7.0, and a phylogenetic tree was constructed (Figure 5). The results demonstrated that these genes clustered into three distinct clades: Clade II contained fabF and ACOT, while Clade III comprised ECR, KCR, fabZ, and KCS. Clade I, the largest group, was further divided into two subclades: Subclade I included ACACA (Acetyl−CoA Carboxylase Alpha), accC, accA, ACSL, fabH (β−Ketoacyl−ACP Synthase III), HCD, FATA, and FATB (Acyl−ACP Thioesterase B), whereas Subclade II consisted of fabI, fabD, and fabG.

Cis−acting element profiling in promoter regions: To elucidate the potential roles of wax−related long−chain−fatty−acid metabolism genes in environmental adaptation, 2.0 kb promoter regions of the 17 genes were analyzed. A total of 22 cis−acting elements were identified across these promoters. Notably, all 17 genes contained both light−responsive and common cis−acting elements in promoter and enhancer regions, indicating universal light regulation. The predicted hormone−responsive elements were differentially distributed: an abscisic acid (ABA)−responsive element was detected in 12 genes (*KCS*, *ECR*, *HCD*, *FATA*, *fabG*, *KCR*, *accA*, *fabI*, *fabF*, *ACACA*, *ACOT*, and *ACSL*); an auxin−responsive element was present in 10 genes (*accC*, *HCD*, *fabG*, *KCR*, *accA*, *FATB*, *ACOT*, *ECR*, *fabH*, and *ACACA*); a gibberellin−responsive element was identified in 8 genes (*fabD*, *ECR*, *fabG*, *KCS*, *accA*, *fabI*, *FATB*, and *ACSL*); salicylic acid (SA)−responsive elements were observed in 6 genes (*accC*, *HCD*, *accA*, *fabI*, *ACOT*, and *ACSL*); and MeJA−responsive elements were found in the *HCD*, *fabG*, *KCR*, *accA*, *FATB*, and *fabF* genes. Strikingly, accA possessed cis−acting elements responsive to five phytohormones (ABA, auxin, gibberellin, SA, and MeJA), highlighting its potential regulatory complexity.

### 3.6. Transcriptional Regulation of Wax Fatty Acid Metabolism Genes in Response to Phytohormone Treatments

**Figure 6:**
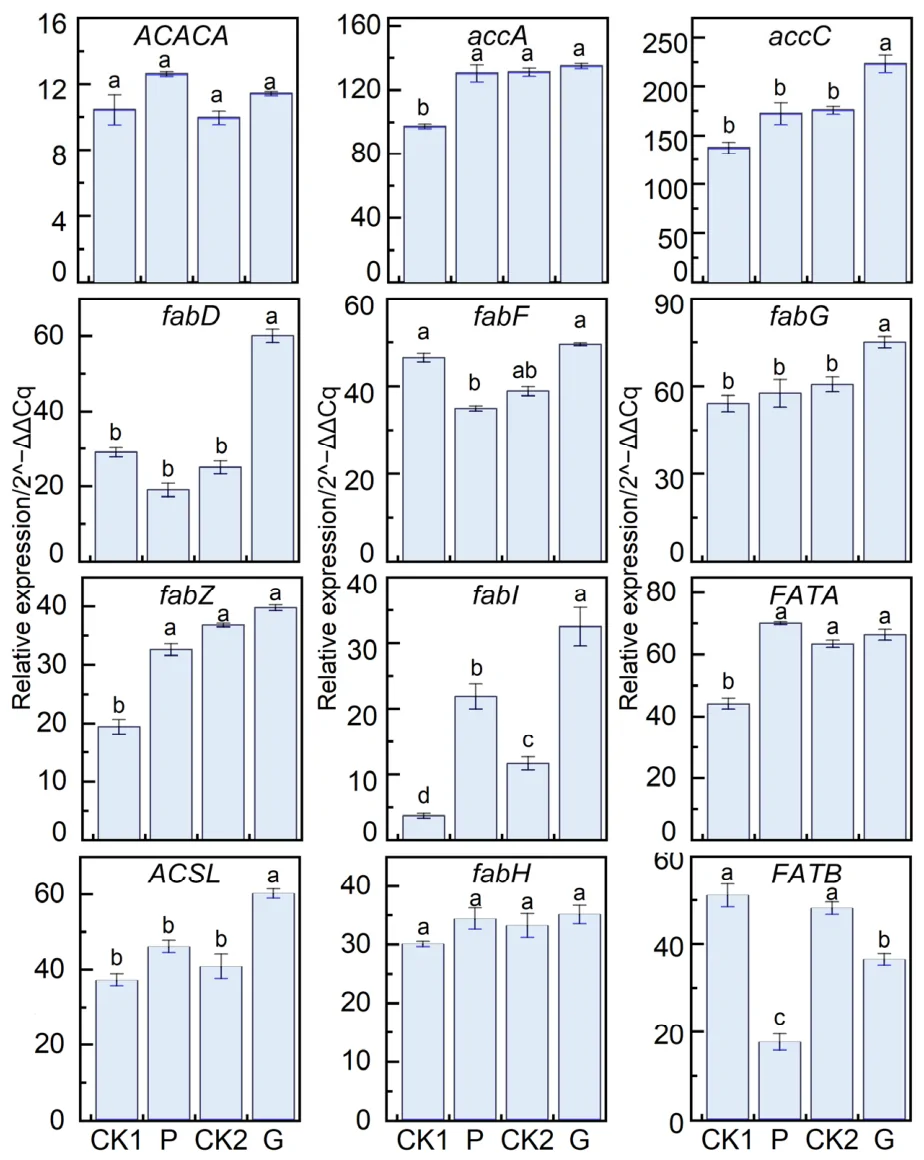
Changes in the expression levels of genes related to fatty acid biosynthesis after treatment with gibberellin and paclobutrazol. Data are presented as means ± SDs (*n* = 3 biological replicates). Different lowercase letters indicate significant differences among treatments determined via one−way ANOVA followed by Duncan’s multiple−range test (*p* < 0.05).

**Figure 7:**
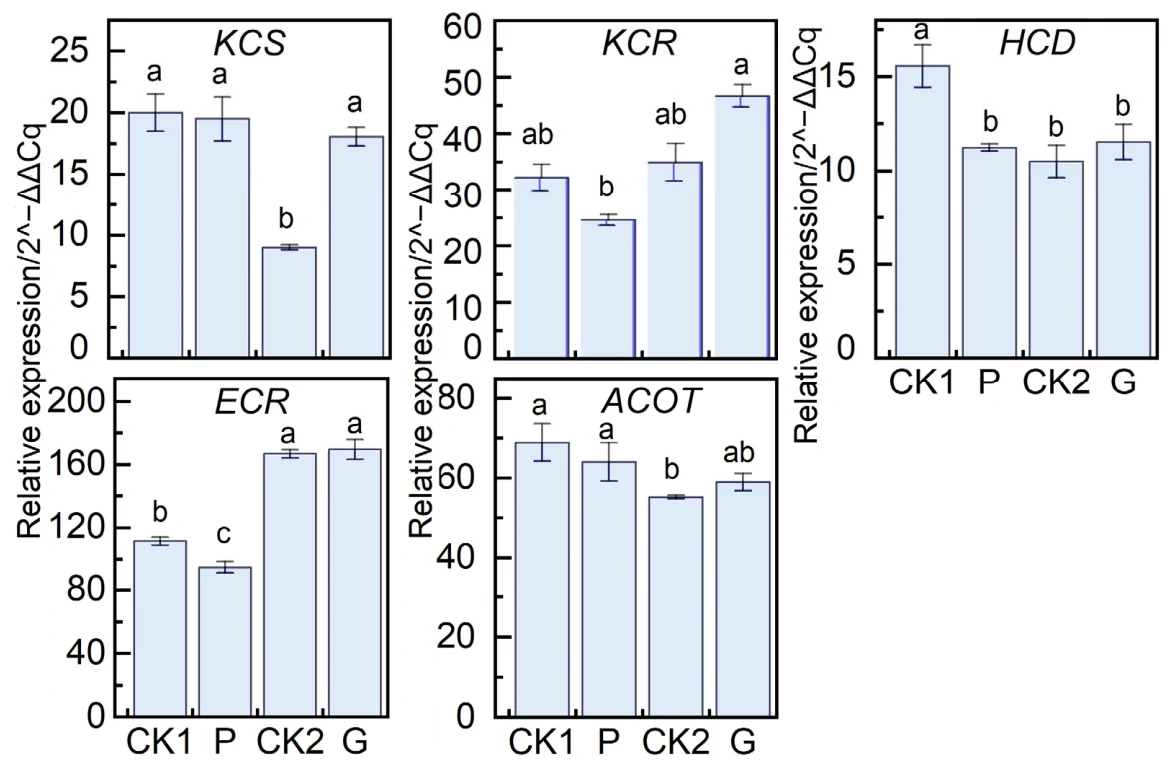
Changes in the expression levels of fatty−acid−elongation−related genes after treatment with gibberellin and paclobutrazol. Data are presented as means ± SDs (*n* = 3 biological replicates). Different lowercase letters indicate significant differences among treatments determined via one−way ANOVA followed by Duncan’s multiple−range test (*p* < 0.05).

Using the prediction results regarding the promoters of genes related to fatty acid metabolism in jujube wax, we studied the expression levels of genes related to fatty acid metabolism in jujube wax after spraying the plants with the plant growth regulators paclobutrazol and gibberellin. The results showed that after treatment with paclobutrazol, the ripening period for ‘Huping jujube’ fruit was about 10 days earlier than that after treatment with gibberellin. In the late stage of fruit development, the expression levels of *accA*, *fabZ*, *fabI*, *FATA*, and *ECR* genes significantly increased (Figure 6 and Figure 7). The expression levels of *KCS*, *HCD*, and *ACOT* genes decreased significantly. This is consistent with the changes in transcriptome gene expression levels shown in Figure 3 and Figure 4. After the plants were sprayed with paclobutrazol, the gene expression levels of *accA*, *fabZ*, *fabI*, and *FATA* were significantly higher than those in the control group; the expression levels of *fabF*, *FATB*, *HCD*, and *ECR* genes were significantly lower. After the plants were sprayed with gibberellin, the gene expression levels of *accC*, *fabD*, *fabG*, *fabI*, *ACSL*, and *KCS* were significantly higher than those in the control group; the expression levels of the *FATB* gene were significantly lower.

### 3.7. Changes in the Waxy Components of Jujubes After Treatment with Plant Growth Regulators

**Figure 8:**
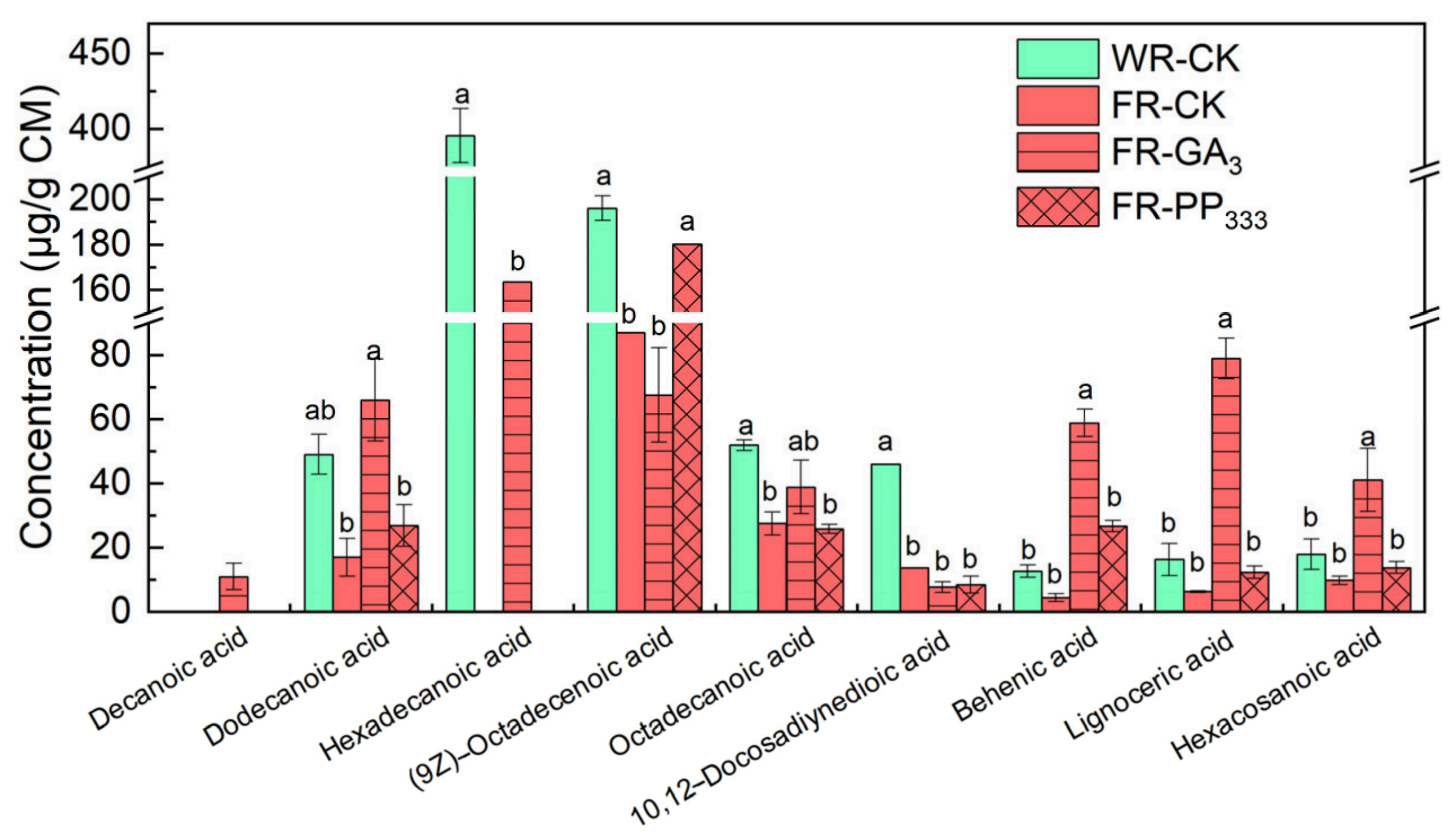
The changes in waxy fatty acids on the surfaces of jujubes after treatment with gibberellin and paclobutrazol. CM: cuticular membrane; WR−CK: white−ripe period control; FR−CK: full−red period control; FR−GA_3_: full−red period, GA_3_−treated; and FR−PP_333_: full−red period, paclobutrazol−treated. Data are presented as means ± SDs (*n* = 3 biological replicates). Different lowercase letters indicate significant differences among treatments determined via one−way ANOVA followed by Duncan’s multiple−range test (*p* < 0.05).

After gibberellin treatment, the content of dodecanoic acid, behenic acid, lignoceric acid, and hexacosanoic acid in the cuticular wax of ‘Hupingzao’ jujubes significantly increased (Figure 8). In contrast, paclobutrazol application markedly increased the content of (9Z)−Octadecenoic acid. Although paclobutrazol is a gibberellin biosynthesis inhibitor, its effects do not correspond directly with those of gibberellin, necessitating further investigation in future studies.

## 4. Discussion

The biosynthesis and transport of plant cuticular waxes involve intricate processes, with numerous enzymes and genes participating in their synthesis and translocation. Our current understanding of wax biosynthesis primarily derives from studies on model species such as Arabidopsis thaliana and Zea mays. To date, more than 190 genes have been found to be implicated in the biosynthesis, transport, and regulation of cuticular waxes [12,32]. These processes can be summarized as three sequential modules: plastidial de novo synthesis of C16/C18 fatty acids [33], ER−localized elongation to very−long−chain fatty acids (VLCFAs, C20–C34) [34], and downstream conversion into alkanes [35], aldehydes, primary alcohols [36], and esters. These modules provide a developmental framework for tracing how plastidial fatty−acid synthesis, ER−mediated VLCFA elongation, and final cuticular−wax composition are coordinated [37]. In our previous study, fatty acids were identified as the most abundant constituents of the cuticular wax on the surfaces of ‘Hupingzao’ jujubes throughout development [22]. A similar pattern of fatty acid accumulation was observed in the cultivars ‘Linhuang No. 1′ (Z. jujuba cv. Linhuang No. 1) and ‘Muzao’ (Z. jujuba cv. Muzao), where the fatty acid concentration gradually increased during fruit development [21]. This study focused on cuticular wax development in ‘Hupingzao’ at both the white−ripe and full−red stages. An integrated transcriptomic and metabolomic analysis was employed to identify key genes and metabolites associated with wax metabolism during fruit development. In this study, genes with *p* < 0.05 and |log2FC| ≥ 1 were screened as differentially expressed genes by comparing the relative expression levels of genes in the white−ripe period and the full−red stage in ‘Hupingzao’ pericarp, and these differentially expressed genes were annotated using the KEGG and GO databases. Three KEGG pathways related to wax metabolism were identified: biosynthesis of secondary metabolites, fatty acid metabolism, and fatty acid degradation. Five GO terms related to the wax metabolism of interest in this study were identified: wax biosynthetic process, fatty acid metabolic process, fruit development, unsaturated fatty acid biosynthetic process, and fatty−acyl−CoA synthase activity (Figure 1D). These findings are consistent with the results reported by Min et al. [38] in regard to the mechanism of wax metabolism during apple development. They compared differences in wax metabolism in apples (Malus domestica Borkh.cv. Red Fuji) at 90, 130, and 150 days after full bloom and identified 11 KEGG pathways, including fatty acid biosynthesis, fatty acid degradation, and fatty acid elongation. A total of 163 differentially expressed genes were identified.

To construct a pathway−level interpretation, we integrated the metabolomic data with KEGG enrichment analysis, following a strategy similar to that of Lang et al. [31], who used LC−MS−based metabolomics combined with KEGG enrichment and network analysis to link differential metabolites to specific biochemical pathways. In our study, 257 of the 530 metabolites were annotated to KEGG pathways, and fatty−acid biosynthesis and fatty−acid elongation were selected for further analysis. In this study, molecular metabolic maps of the substances during the white−ripe (WR) and full−red (FR) periods in jujube (Ziziphus jujuba Mill. ‘Hupingzao’) fruits were constructed using non−targeted metabolomics technology. A total of 530 metabolites were identified, covering 16 major metabolic categories, such as hydrocarbons, lignans, neolignans and related compounds, alkaloids and derivatives, and lipids and lipid−like molecules. Lipids and lipid−like molecules were the most abundant metabolic components, containing 204 metabolites, including fatty acyls, glycerolipids, glycerophospholipids, and other branches. These substances provide crucial precursor materials for the synthesis of waxes. In total, 257 of the 530 metabolites were annotated to KEGG pathways, and two waxy metabolic pathways—fatty acid biosynthesis and fatty acid elongation—were selected for further analysis through enrichment analysis. This study focused on the expression of related genes and the changes in the metabolites of these two metabolic pathways. The pericarp wax components changed significantly during the development of the ‘Hupingzao’ fruit. The total content of fatty acids during the white−ripe period and the relative abundances of various free fatty acids (such as hexadecanoic acid (C16:0), stearic acid (C18:0), oleic acid ((9Z)−octadecenoic acid), arachidic acid (C20:0), lignoceric acid (C24:0), and hexacosanoic acid (C26:0)) were significantly higher than those during the full−red period. This phenomenon reflects the dynamic trend of wax synthesis in fruits: The white−ripe period is a crucial period for the large−scale synthesis and accumulation of fatty acids (especially very−long−chain fatty acids), which are necessary to meet the rapidly expanding fruit pericarp’s demand for barrier substances. Although the total wax may continue to be deposited during the full−red period, the relative content of free fatty acids reduced because they were preferentially converted into other derivatives (alkanes, esters, etc.) or integrated into the cuticle. A similar trend has also been reported in developing apples—i.e., a higher proportion of fatty acids in young fruit and a higher proportion of alkanes and esters in the late fruit period—and it is considered to be the common developmental pattern of wax metabolism in most fruits [38].

Differences in the expression of genes related to wax long−chain−fatty−acid metabolism in fruit will lead to differences in wax composition [39]. In this study, the gene expression of the fatty acid de novo synthesis pathway was not synchronized with the accumulation of metabolites, while the transcriptional differences of the fatty acid elongation pathway were highly consistent with the accumulation of very−long−chain fatty acids. In this study, the transcriptional levels of most genes related to fatty acid synthesis (such as *accA*, *fabG*, *fabZ*, *fabI*, *FATA*, and *ACSL*) were actually higher during the full−red period than during the white−ripe period. This ‘transcription increase and product decrease’ phenomenon may result from feedback regulation or changes in metabolic flux. The downstream extension and modification pathways of wax metabolism during the full−red period accelerate the rate of consumption of precursors, prompting cells to upregulate the expression of synthesis genes to compensate for the substrate consumption. However, because of other competing pathways (such as respiration and energy metabolism) or enzyme activity limitations, the steady−state level of free fatty acids still shows a downward trend. Notably, the expression levels of *fabF* (*KAS II*, responsible for the C16→C18 extension) were significantly higher during the white−ripe period relative to the full−red ripening period. This finding is completely consistent with the high accumulation of stearic and oleic acid during the white−ripe period, indicating that fabF may be the key node in determining C18 fatty acid production. These data directly support an association between *fabF* expression and C18 fatty−acid accumulation, but they do not establish causality. The enzymatic role of fabF in C16→C18 elongation is supported by previous experimental studies [40,41], whereas its regulatory contribution in this study remains to be validated. 

Contrary to the expression pattern of de novo synthetic genes, the expression levels of Fatty Acid Elongase (FAE), including the *KCS* (rate−limiting enzyme), *KCR*, and *HCD* genes involved in endoplasmic reticulum fatty acid elongation, were significantly higher during the white−ripe period relative to the full−red period. This corresponds to the enrichment of very−long−chain fatty acids (C20−C26) during the white−ripe period. The expression levels of the encoding genes (such as FAE1 and the *KCS* family) and substrate specificity directly determine the carbon chain length and content of VLCFAs [42]. The higher expression of *KCS* during the white−ripe period increased the flux in the synthesis of VLCFAs, providing sufficient precursors for the subsequent generation of waxy components. This result is consistent with the decrease in total wax content caused by the downregulation of *KCS* in the onion wax deletion mutant glo1 and emphasizes the rate−limiting role played by KCS in wax accumulation [43]. In one study, overexpression of the FAE1 gene in Arabidopsis significantly increased the content of VLCFAs such as C20:1 and C22:1 in seeds. On the contrary, the synthesis of VLCFAs in FAE1 gene deletion mutants was blocked [44]. During the fruit development in Xanthocras sorbifolium, genes such as *KCS* and *KCR* were highly expressed during the expansion period, which was synchronized with the increased demand for VLCFAs [45]. Furthermore, the high expression of *ECR* during the full−red period may reflect its unique function in adjusting the length of the substrate chain or be related to its preference for specific wax components (such as alcohols and esters). Genetic studies in Arabidopsis have demonstrated that KCS1, KCS2, KCS6/CER6/CUT1, and KCS9 are required for the biosynthesis of specific cuticular−wax components [46], providing experimental support for the function of KCS in wax biosynthesis. In our dataset, the coordinated expression of *KCS* with VLCFA accumulation is correlative, and the causal role of KCS in this system remains a testable hypothesis.

Analysis of gene promoter elements provides a basis for exploring potential hormone regulation. We found that hormone response elements such as ABA, GA, IAA, MeJA, and SA were widely distributed in the promoter regions of 17 fatty acid metabolism genes. The synthesis of plant cuticular wax was complexly regulated by light, temperature, moisture, and various hormones. The promoters of these core transcription factors (such as MYB, SHN/WIN1, and ERF) also contain an abundance of hormone−responsive elements. These transcription factors bind to the promoters of structural genes such as *KCS* and *ECR* to carry out regulatory instructions [39]. We found that the application of gibberellin GA_3_ was associated with significant upregulation of genes involved in fatty acid biosynthesis and elongation, such as *accC*, *fabD*, *fabG*, *fabI*, *ACSL*, and *KCS*, while downregulating the expression of *FATB* (which preferentially releases saturated fatty acids). GA_3_ treatment also increased the content of dodecanoic acid, behenic acid, lignoceric acid, and hexacosanoic acid in the waxes. These results are consistent with the physiological role of GA in promoting cell elongation and fruit enlargement, but they do not by themselves demonstrate that GA directly regulates wax synthesis genes to match the rapid increase in surface area. In contrast, paclobutrazol (a gibberellin inhibitor) treatment was associated with downregulation of *KCS*, *HCD*, and *ACOT*, as well as regulation of genes such as *accA*, *fabZ*, *fabI*, and *FATA*, and specifically increased the content of oleic acid ((9Z)−octadecenoic acid). This might be related to the distinct effects of GA signaling on the two modules of fatty acid ‘de novo synthesis’ and ‘elongation’. When GA signaling was weakened, the elongation pathway appeared to be restricted, and the C18:1 synthesized by plastids could not be efficiently elongated. Notably, the GA_3_ and PP_333_ treatments may have altered developmental timing and fruit growth. Some of the effects on wax−related genes and metabolites may reflect developmental differences rather than direct hormonal regulation of wax metabolism. Endogenous GA levels were not measured in this study, and future work should quantify GA levels and use physiological developmental staging to separate direct hormonal effects from indirect developmental effects. The upregulation of *FATA* further promoted the hydrolysis and accumulation of oleic acid from ACP. Similarly, exogenous ABA and MeJA treatments were also found to significantly affect the expression of wax−related genes and change the mechanical strength of the fruit [47]. The changes in gene expression induced by hormone treatment in ‘Hupingzao’ may be achieved by activating homologous transcription factors (such as ZjMYB96 and ZjSHN). In other systems, ABA and JA act synergistically to promote cuticular wax biosynthesis, and the ABA−responsive transcription factor MYB96 directly binds to the promoters of KCS1, KCS2, KCS6, and KCR1 to enhance VLCFA elongation and wax deposition [48]. These findings provide a mechanistic template for interpreting our hormonal data, but direct hormonal regulation of *fabF* or *KCS* in our system has not been experimentally confirmed and is therefore presented as a hypothesis. This hypothesis requires verification by subsequent yeast one−hybrid or ChIP−seq experiments.

In this study, the transcriptomic and metabolomic changes associated with cuticular wax metabolism during the development of jujube (Ziziphus jujuba Mill. ‘Hupingzao’) fruit from the white−ripe to full−red periods were characterized. The high expression of the *fabF* gene (*KASII*, catalyzing C16→C18 extension) in the de novo synthesis stage of fatty acids was highly consistent with the accumulation of C18 fatty acids. During the fatty acid elongation stage, *KCS*, as well as *KCR* and *HCD*, showed significantly high expression in the white−ripe period, directly driving the synthesis of very−long−chain fatty acids. These results are consistent with the proposed role of *KCS* as an important node in regulating VLCFA yield in ‘Hupingzao’, but the causal relationship remains to be validated. The exogenous hormone treatment experiments suggested that GA treatment was associated with changes in the balance between the ‘synthesis’ and ‘elongation’ modules of fatty acids. We propose an evidence−graded working model. First, in plastids, our data directly support an association between high *fabF* expression at the white−ripe period and C18 fatty−acid accumulation; causality remains to be tested. Second, in the endoplasmic reticulum, previous genetic studies experimentally support KCS as a rate−limiting enzyme in VLCFA elongation [40], while our data provide correlative support for the coordinated expression of *KCS*, *KCR*, and *HCD* with VLCFA (C20–C26) enrichment. Third, in downstream wax formation, our data support VLCFA enrichment at the white−ripe stage and a shift toward alkanes, aldehydes, primary alcohols, and esters at the full−red stage; this conversion is a biologically plausible interpretation based on established literature. Fourth, hormonal regulation by GA/ABA/JA is supported by external experimental evidence [48], but in our system it remains a hypothesis because no signaling pathway validation was performed. A testable prediction of this model is that manipulation of *KCS* expression or hormonal treatment would lead to corresponding changes in VLCFA profiles and wax composition. Future studies using GA signaling mutants, yeast one−hybrid, ChIP−seq, or other functional assays are needed to test whether GA directly regulates wax biosynthesis. In a broader structure–function context, Li et al. showed that macromolecular domain architecture can determine biological activity [49]. Although their system is not directly related to cuticular wax, this example illustrates that metabolite function may depend not only on abundance but also on structural integration. This citation is therefore intended only as a conceptual reference, not as mechanistic support for the wax pathway. 

## Data Availability

The data presented in this study are available from the corresponding author on request to ensure privacy.

## Supplementary Materials

The following supporting information can be downloaded at https://www.mdpi.com/article/10.3390/life16101685/s1, Table S1: Informations and Primers of genes. Table S2: Raw data quality control. Table S3: GO Enrichment. Table S4: KEGG enrichment. Table S5: The proportional composition of wax components in ‘Hupingzao’ jujube fruits.

## Abbreviations

The following abbreviations are used in this manuscript:

GC−MS	gas chromatography–mass spectrometry
LCFAs	long−chain fatty acids
GA_3_	gibberellic acid
PP_333_	paclobutrazol
DEGs	differentially expressed genes
KEGG	Kyoto Encyclopedia of Genes and Genomes
FPKM	Fragments per kilobase million
HIS	Histon−H
VLCFAs	very−long−chain fatty acid
ACACA	acetyl−CoA carboxylase alpha
accA	acetyl−CoA carboxylase, carboxyltransferase subunit alpha
accC	acetyl−CoA carboxylase, biotin carboxylase subunit
fabD	Malonyl−CoA:ACP transacylase
fabF	β−Ketoacyl−ACP synthase II
fabG	β−Ketoacyl−ACP reductase
fabZ	β−Hydroxyacyl−ACP dehydratase
fabI	enoyl−ACP reductase
FATA	acyl−ACP Thioesterase A
ACSL	long−chain acyl−CoA synthetase
fabH	β−Ketoacyl−ACP synthase III
FATB	acyl−ACP thioesterase B
KCS	β−ketoacyl−CoA synthase
KCR	β−ketoacyl−CoA reductase
HCD	β−hydroxyacyl−CoA dehydratase
ECR	enoyl−CoA reductase
ACOT	acyl−CoA thioesterase
