# Supplementary material for: Integrated Transcriptomic and Metabolomic Analyses Reveal the Regulatory Networks of Cuticular Wax Fatty Acid Metabolism in Jujube (Ziziphus jujuba Mill. ‘Hupingzao’)
Source: Life (Basel). 2026 Oct 9;16(10):1685. doi: 10.3390/life16101685 (PMC13706060; doi:10.3390/life16101685)
Supplement: Supplementary file 1 [file life-16-01685-s001.zip › Table S1.pdf]

Table S1 Informations and Primers of genes

Gene symbol	Protein name	Jujube gene ID (PRJNA1014986)	Closest ortholog (UniProtKB/Swiss-Prot)	% identity	E-value	GenBank accession (if cloned)	Forward	R
ACACA	Acetyl-CoA Carboxylase Alpha	LOC107420964	ACC1 — Arabidopsis thaliana	82%	0.0	XM_048476369.1	AGGGATCCTTCAGGCTGGAT	GACTGTCCACGACAACCCAT
accA	Acetyl-CoA Carboxylase, Carboxyltransferase Subunit Alpha	LOC107430748	ACCA — Arabidopsis thaliana	62%	0.0	XM_016041626.3	GAAGCTGCCAACATTTGCCA	GCCGTTCCAACACCTCCTTA
accC	Acetyl-CoA Carboxylase, Biotin Carboxylase Subunit	LOC107417663	ACCC2 — Populus trichocarpa	90%	0.0	XM_016026287.3	TGCGCATGAGATGGGGATAC	CACCAATGCAAACCGCTTCA
fabD	Malonyl-CoA:ACP Transacylase	LOC107411556	FABD — Mus musculus	43%	2e-67	XM_016019164.3	GCACCAGCTACTCCACCATT	AGGAGCTCATGGTGGCTAGA
fabF	β-Ketoacyl-ACP Synthase II	LOC107434672	KASC2 — Arabidopsis thaliana	64%	0.0	XM_048473584.1	TGGATCTGGGATGGATGGGT	GCATCATGTGTGCTTCACCC
fabG	β-Ketoacyl-ACP Reductase	LOC107428383	FABG — Cuphea lanceolata	76%	1e-165	XM_016038916.3	GCATCTGGTGGACAAGCTCT	TCCACAGTTCCCCAAGCATC
fabZ	β-Hydroxyacyl-ACP Dehydratase	LOC107427465	FABZ — Synechocystis sp. (strain ATCC 27184 / PCC 6803 / Kazusa)	56%	2e-49	XM_016037835.3	GATTGCTGGCGACACATTGG	CTCGCATACAACCTCCCCTC
fabI	Enoyl-ACP Reductase	LOC107432338	FABI — Arabidopsis thaliana	71%	0.0	XM_016043458.3	TGGAGCAGCCCATAACGATC	GTTCAATGCTGGCACCCAAG
FATA	Acyl-ACP Thioesterase A	LOC107426715	FATA1 — Arabidopsis thaliana	70%	0.0	XM_016036962.3	TTCACTCGACGGAAAGACGG	ACCTCAACCGATCTGCCAAG
ACSL	Long-chain Acyl-CoA Synthetase	LOC107435779	CCL6 — Humulus lupulus	81%	0.0	XM_048480635.1	TGAAAGCTGTGGTGGGTGTT	TTCAGGAACCGACTCAAGCC
fabH	β-Ketoacyl-ACP Synthase III	LOC107434189	FABH2 — Cuphea wrightii	79%	0.0	XM_016045622.3	TGCTTGCCACATTAGGGGAG	TGCACGTTCCTCTGTCAGTC
FATB	Acyl-ACP Thioesterase B	LOC107435376	FATB — Arabidopsis thaliana	75%	0.0	XR_001583007.3	AGCTTGACAATGAGACCGCA	ATTGCCAGTGGAGCACTCTC
KCS	β-ketoacyl-CoA synthase	LOC107403372	KCS4 — Arabidopsis thaliana	85%	0.0	XM_016010249.3	GGTGCTGCTGTTCTGCTTTC	AATGGCCATGAGGTCCTTGG
KCR	β-ketoacyl-CoA reductase	LOC107428452	KCR1 — Arabidopsis thaliana	67%	2e-160	XM_016038979.3	TCAGAGGATTACCGAGGCCA	GCATCCCAGGAAGAACAGCT
HCD	β-hydroxyacyl-CoA dehydratase	LOC107425142	HACDA — Oryza sativa subsp. japonica	80%	3e-132	XM_016035081.3	TGTCAGGCGTCTCTACCTCA	GAATCTCCAAAACGGCAGCG
ECR	enoyl-CoA reductase	LOC107413483	TECR — Arabidopsis thaliana	79%	0.0	XM_016021439.3	CGTCGTTTCTCGTAGCGGTA	CTCCTGCAGATCAGCTACCG
ACOT	Acyl-CoA Thioesterase	LOC107404880	ACH2 — Arabidopsis thaliana	70%	0.0	XM_048477895.1	TCGAGTTCTTGAGCTGCGTT	CTCCAGTCTCTCCCTCACGA
