# Supplementary material for: Integrated Transcriptomic and Metabolomic Analyses Reveal the Regulatory Networks of Cuticular Wax Fatty Acid Metabolism in Jujube (Ziziphus jujuba Mill. ‘Hupingzao’)
Source: Life (Basel). 2026 Oct 9;16(10):1685. doi: 10.3390/life16101685 (PMC13706060; doi:10.3390/life16101685)
Supplement: Supplementary file 1 [file life-16-01685-s001.zip › Table S2.pdf]

Table S2 Raw data quality control

Sample	Raw reads	Clean reads	Q20%	Q30%	GC content%
White-ripe period	33738016	26342474	99.22	96.23	45
Full-red period	54472066	52005642	99.30	96.02	45
