# Supplementary material for: Integrated Transcriptomic and Metabolomic Analyses Reveal the Regulatory Networks of Cuticular Wax Fatty Acid Metabolism in Jujube (Ziziphus jujuba Mill. ‘Hupingzao’)
Source: Life (Basel). 2026 Oct 9;16(10):1685. doi: 10.3390/life16101685 (PMC13706060; doi:10.3390/life16101685)
Supplement: Supplementary file 1 [file life-16-01685-s001.zip › Table S3.pdf]

Table S3 GO Enrichment

GO term	Significant ID number	Up	Down	Background ID number	Rich factor	P value	Significant p value	Maped ID number	GO category
translation	112	95	17	275	0.4073	1.39137E-11	****	256	
response to hydrogen peroxide	35	9	26	74	0.473	2.71172E-06	****	69	
starch catabolic process	16	12	4	25	0.64	1.1538E-05	****	24	
response to karrikin	32	17	15	70	0.4571	1.72682E-05	****	62	
response to high light intensity	35	11	24	82	0.4268	4.34402E-05	****	74	
response to cold	84	47	37	250	0.336	4.85485E-05	****	218	
response to heat	61	23	38	169	0.3609	5.10813E-05	****	150	
phospholipid biosynthetic process	12	7	5	18	0.6667	8.41227E-05	****	18	
response to salt stress	114	58	56	369	0.3089	0.000152101	***	326	
response to abscisic acid	104	56	48	332	0.3133	0.000162718	***	286	
iron-sulfur cluster assembly	16	3	13	30	0.5333	0.000251807	***	29	
response to virus	22	12	10	48	0.4583	0.00033101	***	44	
cellular response to oxidative stress	22	16	6	48	0.4583	0.00033101	***	45	
obsolete oxidation-reduction process	226	119	107	816	0.277	0.000382071	***	685	
cytoplasmic translation	21	21	0	47	0.4468	0.000683785	***	45	
trehalose metabolism in response to stress	7	1	6	9	0.7778	0.000713634	***	9	
response to starvation	7	1	6	9	0.7778	0.000713634	***	9	
positive regulation of transcription elongation by RNA polymerase II	7	1	6	9	0.7778	0.000713634	***	9	
response to cadmium ion	87	32	55	281	0.3096	0.00079599	***	249	
cellular response to phosphate starvation	15	9	6	30	0.5	0.000955692	***	29	
response to toxic substance	11	7	4	19	0.5789	0.000967243	***	18	
starch biosynthetic process	17	13	4	36	0.4722	0.001018865	**	35	
glutathione catabolic process	9	2	7	14	0.6429	0.001019734	**	14	
response to temperature stimulus	13	4	9	25	0.52	0.001308222	**	24	
hyperosmotic salinity response	25	18	7	62	0.4032	0.001394088	**	52	
heat acclimation	10	4	6	17	0.5882	0.001404831	**	17	
nucleotide-sugar metabolic process	10	3	7	17	0.5882	0.001404831	**	16	
protein folding	64	29	35	200	0.32	0.001497592	**	185	
wax biosynthetic process	11	8	3	20	0.55	0.001710414	**	19	
cellular response to heat	12	8	4	23	0.5217	0.001931824	**	20	
response to water deprivation	76	43	33	247	0.3077	0.001968109	**	223	
auxin metabolic process	9	7	2	15	0.6	0.002036684	**	14	
cell redox homeostasis	39	24	15	112	0.3482	0.002279279	**	104	
glucose import	21	11	10	51	0.4118	0.002410807	**	34	
fatty acid metabolic process	21	6	15	51	0.4118	0.002410807	**	40	
drought recovery	6	3	3	8	0.75	0.002474181	**	7	
photosynthetic electron transport in photosystem II	4	4	0	4	1	0.002645399	**	4	
xanthophyll metabolic process	4	2	2	4	1	0.002645399	**	4	
regulation of ruffle assembly	4	0	4	4	1	0.002645399	**	4	

positive regulation of post-transcriptional gene silencing	5	5	0	6	0.8333	0.002918689	**	6	Biological Process
chlorophyll metabolic process	5	1	4	6	0.8333	0.002918689	**	6	
xanthophyll biosynthetic process	5	4	1	6	0.8333	0.002918689	**	6	
protein maturation by iron-sulfur cluster transfer	5	1	4	6	0.8333	0.002918689	**	6	
defense response to fungus	17	3	14	39	0.4359	0.00298992	**	33	
ribosomal large subunit assembly	17	16	1	39	0.4359	0.00298992	**	37	
auxin biosynthetic process	9	4	5	16	0.5625	0.003722046	**	15	
photosynthesis, light harvesting in photosystem I	9	4	5	16	0.5625	0.003722046	**	16	
circadian rhythm	27	11	16	73	0.3699	0.003988454	**	65	
reductive pentose-phosphate cycle	7	7	0	11	0.6364	0.004225524	**	11	
carbohydrate biosynthetic process	14	9	5	31	0.4516	0.00464402	**	31	
response to stress	18	10	8	44	0.4091	0.005206797	**	42	
vitamin E biosynthetic process	6	4	2	9	0.6667	0.005995275	**	8	
shade avoidance	6	3	3	9	0.6667	0.005995275	**	7	
response to desiccation	9	7	2	17	0.5294	0.006328912	**	14	
glucose metabolic process	9	4	5	17	0.5294	0.006328912	**	16	
trehalose biosynthetic process	9	1	8	17	0.5294	0.006328912	**	16	
ribosomal large subunit biogenesis	9	8	1	17	0.5294	0.006328912	**	17	
response to wounding	58	35	23	189	0.3069	0.006607512	**	153	
regulation of defense response	21	16	5	55	0.3818	0.006887747	**	47	
abscisic acid biosynthetic process	7	6	1	12	0.5833	0.008163644	**	11	
mannan catabolic process	5	4	1	7	0.7143	0.008298516	**	5	
endonucleolytic cleavage in ITS1 to separate SSU-rRNA from 5.8S rRNA and LSU-rRNA from tricistronic rRNA transcript (SSU-rRNA, 5.8S rRNA, LSU-rRNA)	5	4	1	7	0.7143	0.008298516	**	7	
response to unfolded protein	8	4	4	15	0.5333	0.009472738	**	14	
chloroplast fission	9	8	1	18	0.5	0.010137074	*	16	
lateral root development	9	6	3	18	0.5	0.010137074	*	16	
monoatomic ion transmembrane transport	16	12	4	40	0.4	0.010517557	*	31	
abscisic acid-activated signaling pathway involved in stomatal movement	4	0	4	5	0.8	0.010828047	*	5	
endonucleolytic cleavage to generate mature 3'-end of SSU-rRNA from (SSU-rRNA, 5.8S rRNA, LSU-rRNA)	4	4	0	5	0.8	0.010828047	*	5	
chlorophyll biosynthetic process	15	13	2	37	0.4054	0.011362155	*	35	
sulfate assimilation	15	12	3	37	0.4054	0.011362155	*	35	
putrescine biosynthetic process from arginine	3	2	1	3	1	0.011667026	*	3	
photosystem II oxygen evolving complex assembly	3	3	0	3	1	0.011667026	*	3	
negative regulation of unidimensional cell growth	3	2	1	3	1	0.011667026	*	3	
positive regulation of microtubule depolymerization	3	2	1	3	1	0.011667026	*	3	
methionine metabolic process	3	0	3	3	1	0.011667026	*	3	
actin filament-based process	3	2	1	3	1	0.011667026	*	3	
positive regulation of protein catabolic process	3	1	2	3	1	0.011667026	*	3	
response to copper ion	3	1	2	3	1	0.011667026	*	3	

negative regulation of organ growth	3	2	1	3	1	0.011667026	*	3
spermatogenesis	3	0	3	3	1	0.011667026	*	3
regulation of DNA methylation-dependent heterochromatin	3	1	2	3	1	0.011667026	*	3
plastid transcription	6	3	3	10	0.6	0.012121923	*	10
protein dephosphorylation	30	18	12	90	0.3333	0.01344673	*	84
response to ozone	11	8	3	25	0.44	0.014623608	*	20
syncytium formation	8	5	3	16	0.5	0.015223429	*	15
post-transcriptional gene silencing	8	7	1	16	0.5	0.015223429	*	16
defense response to virus	14	8	6	35	0.4	0.01620365	*	29
regulation of seed dormancy process	5	4	1	8	0.625	0.018005742	*	8
response to iron ion starvation	5	1	4	8	0.625	0.018005742	*	7
siRNA processing	15	11	4	39	0.3846	0.019259248	*	30
jasmonic acid biosynthetic process	14	6	8	36	0.3889	0.021100218	*	28
mucilage biosynthetic process	6	2	4	11	0.5455	0.021597602	*	10
protein refolding	9	2	7	20	0.45	0.022519567	*	19
obsolete negative regulation of nucleic acid-templated transcription	7	7	0	14	0.5	0.023002462	*	14
systemic acquired resistance	13	7	6	33	0.3939	0.023070338	*	29
post-embryonic development	13	10	3	33	0.3939	0.023070338	*	30
positive regulation of seed germination	8	2	6	17	0.4706	0.023129667	*	16
regulation of stomatal closure	8	4	4	17	0.4706	0.023129667	*	16
xenobiotic metabolic process	4	1	3	6	0.6667	0.026646763	*	6
L-phenylalanine biosynthetic process	4	2	2	6	0.6667	0.026646763	*	6
cellular response to unfolded protein	4	2	2	6	0.6667	0.026646763	*	6
obsolete intracellular metal ion homeostasis	4	0	4	6	0.6667	0.026646763	*	6
pentose-phosphate shunt	4	1	3	6	0.6667	0.026646763	*	6
radial pattern formation	4	3	1	6	0.6667	0.026646763	*	6
cellulose biosynthetic process	18	15	3	51	0.3529	0.02766139	*	43
intracellular water homeostasis	13	9	4	34	0.3824	0.02978468	*	26
protein deubiquitination	13	1	12	34	0.3824	0.02978468	*	34
fruit development	9	7	2	21	0.4286	0.03164675	*	20
photoinhibition	5	2	3	9	0.5556	0.033018824	*	9
cinnamic acid biosynthetic process	5	3	2	9	0.5556	0.033018824	*	8
riboflavin biosynthetic process	5	3	2	9	0.5556	0.033018824	*	9
arginine biosynthetic process	7	7	0	15	0.4667	0.034840971	*	14
cellular response to cold	6	6	0	12	0.5	0.035031559	*	10
response to ethylene	39	22	17	131	0.2977	0.035974223	*	109
response to blue light	11	4	7	28	0.3929	0.036011781	*	23
metabolic process	22	14	8	67	0.3284	0.03671443	*	58
cell population proliferation	13	8	5	35	0.3714	0.037809834	*	31
cellular response to freezing	3	0	3	4	0.75	0.038731905	*	4
protein depalmitoylation	3	3	0	4	0.75	0.038731905	*	4
rRNA export from nucleus	3	3	0	4	0.75	0.038731905	*	4
L-cystine transport	3	1	2	4	0.75	0.038731905	*	4

cellular response to blue light	3	0	3	4	0.75	0.038731905	*	4	
phloem sucrose loading	3	1	2	4	0.75	0.038731905	*	4	
guanosine tetraphosphate metabolic process	3	1	2	4	0.75	0.038731905	*	4	
cellular response to glucose stimulus	3	1	2	4	0.75	0.038731905	*	4	
glycerol-3-phosphate catabolic process	3	2	1	4	0.75	0.038731905	*	4	
tryptophan catabolic process to kynurenine	3	3	0	4	0.75	0.038731905	*	4	
Mo-molybdopterin cofactor biosynthetic process	3	0	3	4	0.75	0.038731905	*	4	
regulation of cell differentiation	3	2	1	4	0.75	0.038731905	*	3	
response to osmotic stress	27	14	13	86	0.314	0.03918466	*	78	
cold acclimation	10	5	5	25	0.4	0.039453655	*	22	
galactose metabolic process	9	3	6	22	0.4091	0.043048772	*	21	
ubiquitin-dependent protein catabolic process	38	7	31	129	0.2946	0.04420606	*	116	
L-ascorbic acid biosynthetic process	8	5	3	19	0.4211	0.04667682	*	14	
unsaturated fatty acid biosynthetic process	8	3	5	19	0.4211	0.04667682	*	15	
polar nucleus fusion	8	3	5	19	0.4211	0.04667682	*	17	
photosystem II assembly	8	7	1	19	0.4211	0.04667682	*	14	
proton motive force-driven ATP synthesis	8	3	5	19	0.4211	0.04667682	*	17	
protein ubiquitination	74	35	39	273	0.2711	0.048373256	*	255	
cytosolic large ribosomal subunit	78	73	5	138	0.5652	8.03732E-18	****	132	Cellular Component
chloroplast	721	445	276	2571	0.2804	1.08991E-11	****	2345	
chloroplast thylakoid	65	46	19	140	0.4643	4.75116E-10	****	135	
chloroplast stroma	167	99	68	481	0.3472	8.8908E-10	****	451	
chloroplast envelope	138	88	50	397	0.3476	2.30482E-08	****	376	
cytosolic small ribosomal subunit	40	40	0	86	0.4651	9.31041E-07	****	80	
thylakoid	54	42	12	135	0.4	4.70666E-06	****	131	
plastoglobule	29	17	12	58	0.5	4.80739E-06	****	56	
vacuole	137	76	61	436	0.3142	1.40832E-05	****	383	
chloroplast thylakoid membrane	99	76	23	305	0.3246	5.2129E-05	****	272	
thylakoid lumen	17	13	4	31	0.5484	0.000102342	***	30	
cytosol	469	229	240	1792	0.2617	0.000169626	***	1604	
cytoplasm	844	411	433	3375	0.2501	0.000310418	***	2835	
plant-type vacuole	25	8	17	57	0.4386	0.000311631	***	53	
chloroplast thylakoid lumen	21	17	4	47	0.4468	0.000683785	***	47	
vacuolar membrane	112	53	59	378	0.2963	0.000962087	***	342	
Golgi apparatus	241	140	101	893	0.2699	0.001201617	**	802	
chloroplast membrane	31	23	8	82	0.378	0.001420598	**	80	
ruffle membrane	4	0	4	4	1	0.002645399	**	4	
ribosome	26	24	2	68	0.3824	0.002763369	**	64	
plasma membrane	99	51	48	339	0.292	0.002967598	**	251	
apoplast	77	48	29	256	0.3008	0.003583097	**	217	
mitochondrial matrix	23	10	13	62	0.371	0.00722228	**	62	
chloroplast inner membrane	26	18	8	74	0.3514	0.009981866	**	70	
euchromatin	4	1	3	5	0.8	0.010828047	*	4	

multivesicular body	4	0	4	5	0.8	0.010828047	*	5
FACT complex	3	0	3	3	1	0.011667026	*	3
pICln-Sm protein complex	3	3	0	3	1	0.011667026	*	3
chloroplast photosystem II	7	6	1	13	0.5385	0.014254652	*	13
cytosolic ribosome	17	13	4	45	0.3778	0.015978245	*	43
obsolete integral component of chloroplast outer membrane	5	2	3	8	0.625	0.018005742	*	8
U1 snRNP	10	7	3	23	0.4348	0.021494865	*	23
peroxisome	50	15	35	169	0.2959	0.022116202	*	152
protein phosphatase type 2A complex	8	6	2	17	0.4706	0.023129667	*	17
mitochondrial large ribosomal subunit	12	12	0	30	0.4	0.025155139	*	25
photosystem I	4	3	1	6	0.6667	0.026646763	*	6
Golgi membrane	64	37	27	227	0.2819	0.03013885	*	204
endosome	73	45	28	263	0.2776	0.030706745	*	235
1,3-beta-D-glucan synthase complex	6	3	3	12	0.5	0.035031559	*	12
SMN-Sm protein complex	3	3	0	4	0.75	0.038731905	*	4
nucleoid	9	7	2	22	0.4091	0.043048772	*	22
RNA polymerase I complex	8	8	0	19	0.4211	0.04667682	*	18
peroxisomal membrane	13	5	8	36	0.3611	0.047254399	*	34
structural constituent of ribosome	151	138	13	347	0.4352	3.70871E-18	****	322
alpha-amylase activity	15	11	4	18	0.8333	8.52264E-08	****	17
pyridoxal phosphate binding	38	19	19	95	0.4	0.000113979	***	82
limit dextrinase activity	8	8	0	10	0.8	0.000200544	***	9
pullulanase activity	8	8	0	10	0.8	0.000200544	***	9
carbohydrate:proton symporter activity	29	13	16	69	0.4203	0.000262677	***	52
alpha,alpha-trehalose-phosphate synthase (UDP-forming) activity	7	1	6	9	0.7778	0.000713634	***	9
small GTPase binding	12	5	7	22	0.5455	0.001162604	**	22
calcium ion binding	58	32	26	177	0.3277	0.001306956	**	153
hydrolase activity	114	65	49	392	0.2908	0.001781262	**	361
glucose transmembrane transporter activity	21	11	10	50	0.42	0.001797631	**	34
alpha-amylase activity	4	0	4	4	1	0.002645399	**	4
siRNA binding	5	4	1	6	0.8333	0.002918689	**	5
trehalose-phosphatase activity	8	0	8	13	0.6154	0.002942781	**	12
2 iron, 2 sulfur cluster binding	16	4	12	36	0.4444	0.003098754	**	35
protein folding chaperone	15	5	10	33	0.4545	0.003176061	**	31
glutathione hydrolase activity	7	2	5	11	0.6364	0.004225524	**	11
hypoglycin A gamma-glutamyl transpeptidase activity	7	2	5	11	0.6364	0.004225524	**	11
RNA-dependent RNA polymerase activity	7	7	0	11	0.6364	0.004225524	**	11
potassium ion transmembrane transporter activity	11	4	7	22	0.5	0.004557384	**	22
protein-folding chaperone binding	13	8	5	28	0.4643	0.004717797	**	28
water channel activity	16	12	4	38	0.4211	0.005918966	**	29
nucleosome binding	6	0	6	9	0.6667	0.005995275	**	9
cysteine-type deubiquitinase activity	14	1	13	32	0.4375	0.006555758	**	32
protein-disulfide reductase activity	25	16	9	69	0.3623	0.00739327	**	62

4 iron, 4 sulfur cluster binding	17	3	14	42	0.4048	0.007405518	**	41	Molecular Function
mannan endo-1,4-beta-mannosidase activity	5	4	1	7	0.7143	0.008298516	**	5	
pigment binding	8	3	5	15	0.5333	0.009472738	**	15	
iron ion binding	24	11	13	67	0.3582	0.010018365	*	61	
GTP binding	48	31	17	155	0.3097	0.010526094	*	142	
GTPase binding	4	2	2	5	0.8	0.010828047	*	5	
sucrose-phosphate synthase activity	4	1	3	5	0.8	0.010828047	*	4	
prephenate dehydratase activity	4	2	2	5	0.8	0.010828047	*	5	
arogenate dehydratase activity	4	2	2	5	0.8	0.010828047	*	5	
phosphatidylinositol-4,5-bisphosphate binding	4	2	2	5	0.8	0.010828047	*	5	
rRNA (adenine-N6,N6-)-dimethyltransferase activity	4	4	0	5	0.8	0.010828047	*	5	
uracil transmembrane transporter activity	3	2	1	3	1	0.011667026	*	3	
GTP-dependent protein binding	3	2	1	3	1	0.011667026	*	3	
myosin XI tail binding	3	2	1	3	1	0.011667026	*	3	
bis(5'-adenosyl)-pentaphosphatase activity	3	1	2	3	1	0.011667026	*	3	
ribose-5-phosphate isomerase activity	3	2	1	3	1	0.011667026	*	3	
cyclase activity	3	0	3	3	1	0.011667026	*	3	
ubiquitin activating enzyme activity	3	1	2	3	1	0.011667026	*	3	
ferrous iron binding	6	1	5	10	0.6	0.012121923	*	10	
phosphatase activity	19	9	10	51	0.3725	0.013209087	*	45	
calmodulin binding	46	27	19	150	0.3067	0.014473558	*	134	
NAD binding	16	9	7	42	0.381	0.017552262	*	40	
L-ascorbate peroxidase activity	5	2	3	8	0.625	0.018005742	*	8	
glycosyltransferase activity	83	46	37	298	0.2785	0.02078324	*	269	
4-coumarate-CoA ligase activity	6	2	4	11	0.5455	0.021597602	*	9	
L-ascorbic acid binding	6	4	2	11	0.5455	0.021597602	*	11	
inorganic phosphate transmembrane transporter activity	6	1	5	11	0.5455	0.021597602	*	9	
transaminase activity	9	5	4	20	0.45	0.022519567	*	18	
N-acetyltransferase activity	7	6	1	14	0.5	0.023002462	*	13	
glutathione gamma-glutamylcysteinyltransferase activity	8	2	6	17	0.4706	0.023129667	*	14	
metal ion binding	279	141	138	1109	0.2516	0.024959727	*	984	
glutathione peroxidase activity	4	1	3	6	0.6667	0.026646763	*	6	
enoyl-CoA hydratase activity	4	0	4	6	0.6667	0.026646763	*	5	
guanosine-3',5'-bis(diphosphate) 3'-diphosphatase activity	4	1	3	6	0.6667	0.026646763	*	6	
obsolete solute:proton antiporter activity	4	4	0	6	0.6667	0.026646763	*	5	
histone binding	14	3	11	37	0.3784	0.027027735	*	36	
glycerol channel activity	13	9	4	34	0.3824	0.02978468	*	26	
alcohol dehydrogenase (NAD+) activity	13	11	2	34	0.3824	0.02978468	*	28	
rRNA binding	19	15	4	55	0.3455	0.030157204	*	52	
oxidoreductase activity, acting on a sulfur group of donors, disulfide as acceptor	15	13	2	41	0.3659	0.030745085	*	38	
NADPH dehydrogenase activity	6	3	3	12	0.5	0.035031559	*	12	
1,3-beta-D-glucan synthase activity	6	3	3	12	0.5	0.035031559	*	12	

catalytic activity	69	37	32	249	0.2771	0.035953736	*	212	
chlorophyll binding	11	5	6	28	0.3929	0.036011781	*	22	
ligase activity	20	11	9	60	0.3333	0.038647681	*	53	
fatty-acyl-CoA synthase activity	3	1	2	4	0.75	0.038731905	*	4	
phosphatidylcholine binding	3	2	1	4	0.75	0.038731905	*	4	
dihydrokaempferol 4-reductase activity	3	1	2	4	0.75	0.038731905	*	4	
flavanone 4-reductase activity	3	1	2	4	0.75	0.038731905	*	4	
palmitoyl-(protein) hydrolase activity	3	3	0	4	0.75	0.038731905	*	4	
L-cystine transmembrane transporter activity	3	1	2	4	0.75	0.038731905	*	4	
adenosine deaminase activity	3	3	0	4	0.75	0.038731905	*	3	
UDP-glucose 4-epimerase activity	3	0	3	4	0.75	0.038731905	*	4	
GTP diphosphokinase activity	3	1	2	4	0.75	0.038731905	*	4	
inorganic cation transmembrane transporter activity	3	3	0	4	0.75	0.038731905	*	4	
glycerol-3-phosphate dehydrogenase [NAD(P)+] activity	3	2	1	4	0.75	0.038731905	*	4	
inositol-polyphosphate 5-phosphatase activity	3	0	3	4	0.75	0.038731905	*	4	
acetyl-CoA C-acetyltransferase activity	3	0	3	4	0.75	0.038731905	*	4	
GTPase activity	49	35	14	171	0.2865	0.0401903	*	143	
obsolete coenzyme binding	18	9	9	53	0.3396	0.040378945	*	43	
ABC-type xenobiotic transporter activity	9	3	6	22	0.4091	0.043048772	*	19	
NADP binding	9	2	7	22	0.4091	0.043048772	*	21	
protein kinase binding	8	6	2	19	0.4211	0.04667682	*	18	
RNA polymerase I activity	8	8	0	19	0.4211	0.04667682	*	18	

Note:Significance levels are indicated by ****, ***, **, and * for p−value ranges of [0.00001, 0.0001),[0.0001, 0.001), [0.001, 0.01), and [0.01, 0.05), respectively.
