# Supplementary material for: Integrated Transcriptomic and Metabolomic Analyses Reveal the Regulatory Networks of Cuticular Wax Fatty Acid Metabolism in Jujube (Ziziphus jujuba Mill. ‘Hupingzao’)
Source: Life (Basel). 2026 Oct 9;16(10):1685. doi: 10.3390/life16101685 (PMC13706060; doi:10.3390/life16101685)
Supplement: Supplementary file 1 [file life-16-01685-s001.zip › Table S4.pdf]

Table S4 KEGG Enrichment

Pathway_ID	KEGG_Level_1	KEGG_Level_2	Pathway_Name	IDs	S	TS	B	TB	Rich.Fac	P.value	Sig.Sign.	Q.value	Sig.Sign.Q	
ko00564	Metabolism	Lipid metabolism	Glycerophospholipid metabolism	C05974, C00670, C00344, C18169, C00416, C05980, C00350, C04230, C04438, C00114, C03819, C01194, C00588, C00641, C00157	15	142	56	6490	0.2679	4.51896E-13	****	9.80614E-11	****	
ko02010	mental Information Pro	Membrane transport	ABC transporters	C00212, C01835, C00009, C00387, C00255, C00062, C00064, C00135, C00079, C00114, C00137, C00315, C00089, C00025, C00492, C00299, C00378, C00049, C00559, C01083	20	142	138	6490	0.1449	1.12914E-11	****	1.22511E-09	****	
ko01060	Metabolism	Chemical structure transformation maps	Biosynthesis of plant secondary metabolites	C00408, C00417, C00712, C00249, C00062, C08491, C00064, C00082, C00135, C00079, C00078, C00152, C00170, C00026, C00254, C00025, C00049, C00158, C01530, C01595	20	142	141	6490	0.1418	1.69705E-11	****	1.22753E-09	****	
ko01100	Metabolism	Global and overview maps	Metabolic pathways	C04593, C01250, C00212, C07215, C03519, C04046, C14825, C00195, C00344, C01825, C02693, C06752, C00408, C00072, C05610, C00416, C05980, C01617, C00805, C00242, C05570, C00954, C01879, C00774, C00402, C00417, C00387, C00864, C06337, C10682, C19569, C02170, C00836, C00249, C00255, C00062, C08491, C00064, C00082, C00497, C00350, C00135, C00079, C09727, C00106, C00146, C03692, C02504, C03546, C00147, C00153, C13508, C00078, C04294, C01190, C00152, C00257, C00114, C05607, C00170, C00958, C05437, C00026, C15767, C00984, C00137, C05581, C00254, C01571, C02183, C00315, C01035, C00089, C02353, C00222, C01040, C00230, C00025, C00492, C00299, C02480, C00388, C01620, C00383, C01194, C00378, C00049, C00588, C05619, C02666, C00559, C06037, C00158, C00641, C01530, C00389, C01595, C00090, C01083, C19615, C14826, C00157, C05580, C01546, C01384, C02679	106	142	3133	6490	0.0338	8.64784E-11	****	4.69145E-09	****	

ko05230	Human Diseases	Cancer: overview	Central carbon metabolism in cancer	C00062, C00064, C00082, C00135, C00079, C00078, C00152, C00026, C00025, C00049, C00158	11	142	37	6490	0.2973	1.96038E-10	****	7.40399E-09	****	
ko04974	Organismal Systems	Digestive system	Protein digestion and absorption	C00062, C00064, C00082, C00135, C00079, C00146, C00078, C00152, C01746, C00025, C00388, C00049	12	142	47	6490	0.2553	2.04719E-10	****	7.40399E-09	****	
ko05231	Human Diseases	Cancer: overview	Choline metabolism in cancer	C00670, C00416, C04230, C00114, C00588, C00157	6	142	11	6490	0.5455	4.1692E-08	****	1.29245E-06	****	
ko01061	Metabolism	Chemical structure transformation maps	Biosynthesis of phenylpropanoids	C05610, C01617, C00805, C00417, C10682, C00079, C09727, C00078, C00026, C00254, C00230, C05619, C02666, C00158	14	142	106	6490	0.1321	5.72049E-08	****	1.55168E-06	****	
ko01230	Metabolism	Global and overview maps	Biosynthesis of amino acids	C01250, C00062, C00064, C00082, C00135, C00079, C02504, C00078, C00152, C00026, C00254, C00025, C00049, C00158	14	142	128	6490	0.1094	6.23475E-07	****	1.50327E-05	****	
ko00970	etic Information Proces	Translation	Aminoacyl-tRNA biosynthesis	C00062, C00064, C00082, C00135, C00079, C00078, C00152, C00025, C00049	9	142	52	6490	0.1731	1.47393E-06	****	3.19842E-05	****	
ko00250	Metabolism	Amino acid metabolism	Alanine, aspartate and glutamate metabolism	C00402, C00064, C00152, C00026, C00025, C00049, C00158	7	142	28	6490	0.25	1.66885E-06	****	3.29219E-05	****	
ko00591	Metabolism	Lipid metabolism	Linoleic acid metabolism	C14825, C14765, C14767, C14829, C01595, C14826, C00157	7	142	29	6490	0.2414	2.15991E-06	****	3.90584E-05	****	
ko00410	Metabolism	Metabolism of other amino acids	beta-Alanine metabolism	C00864, C00135, C00106, C00315, C00222, C00383, C00049	7	142	32	6490	0.2188	4.40886E-06	****	7.35941E-05	****	
ko00220	Metabolism	Amino acid metabolism	Arginine biosynthesis	C01250, C00062, C00064, C00026, C00025, C00049	6	142	23	6490	0.2609	7.33184E-06	****	0.000113643	***	
ko01064	Metabolism	Chemical structure transformation maps	Biosynthesis of alkaloids derived from ornithine, lysine and nicotinic acid	C00408, C00417, C00062, C00079, C05607, C00026, C00025, C00049, C00158	9	142	67	6490	0.1343	1.29634E-05	****	0.000187537	***	
ko01070	Metabolism	Chemical structure transformation maps	Biosynthesis of plant hormones	C00805, C00954, C00417, C08491, C00079, C00078, C00026, C00049, C00158	9	142	68	6490	0.1324	1.46661E-05	****	0.000198908	***	
ko01232	Metabolism	Global and overview maps	Nucleotide metabolism	C00212, C00242, C00387, C00064, C00106, C00147, C00299, C00559	8	142	58	6490	0.1379	3.28074E-05	****	0.000418777	***	
ko00552	Metabolism	Glycan biosynthesis and metabolism	Teichoic acid biosynthesis	C00344, C00114, C00588, C00641	4	142	12	6490	0.3333	9.48763E-05	****	0.001143786	**	
ko00470	Metabolism	Metabolism of other amino acids	D-Amino acid metabolism	C00402, C00062, C00064, C00135, C00079, C00026, C00025, C00049	8	142	69	6490	0.1159	0.00011686	***	0.001334661	**	
ko00561	Metabolism	Lipid metabolism	Glycerolipid metabolism	C04046, C00416, C03692, C13508, C06037, C00641	6	142	38	6490	0.1579	0.000153007	***	0.001660128	**	
ko01210	Metabolism	Global and overview maps	2-Oxocarboxylic acid metabolism	C01250, C00417, C02631, C00082, C00079, C02504, C00078, C00026, C00025, C00049, C00158	11	142	144	6490	0.0764	0.000290048	***	0.002997159	**	
ko04978	Organismal Systems	Digestive system	Mineral absorption	C00009, C00064, C00079, C00078, C00152	5	142	29	6490	0.1724	0.00036337	***	0.003428315	**	
ko04070	mental Information Pro	Signal transduction	Phosphatidylinositol signaling system	C00416, C03546, C00137, C01194, C00641	5	142	29	6490	0.1724	0.00036337	***	0.003428315	**	
ko00340	Metabolism	Amino acid metabolism	Histidine metabolism	C05570, C00135, C00026, C00025, C00388, C00049	6	142	47	6490	0.1277	0.000506291	***	0.00457771	**	

ko04727	Organismal Systems	Nervous system	GABAergic synapse	C00064, C00026, C00025	3	142	9	6490	0.3333	0.000782111	***	0.006788726	**	
ko01065	Metabolism	Chemical structure transformation maps	Biosynthesis of alkaloids derived from histidine and purine	C00417, C00135, C00026, C00388, C00158	5	142	35	6490	0.1429	0.000893946	***	0.007184679	**	
ko00400	Metabolism	Amino acid metabolism	Phenylalanine, tyrosine and tryptophan biosynthesis	C00082, C00079, C00078, C00254, C00230	5	142	35	6490	0.1429	0.000893946	***	0.007184679	**	
ko05034	Human Diseases	Substance dependence	Alcoholism	C00212, C00082, C00025	3	142	10	6490	0.3	0.001099451	**	0.008226929	**	
ko00365	Metabolism	Xenobiotics biodegradation and metabolism	Furfural degradation	C00026, C11101, C01546	3	142	10	6490	0.3	0.001099451	**	0.008226929	**	
ko04138	Cellular Processes	Transport and catabolism	Autophagy - yeast	C00350, C01194	2	142	3	6490	0.6667	0.001405767	**	0.00970072	**	
ko04136	Cellular Processes	Transport and catabolism	Autophagy - other	C00350, C01194	2	142	3	6490	0.6667	0.001405767	**	0.00970072	**	
ko00053	Metabolism	Carbohydrate metabolism	Ascorbate and aldarate metabolism	C01825, C00072, C00026, C00137, C01040, C01620	6	142	57	6490	0.1053	0.001430521	**	0.00970072	**	
ko00940	Metabolism	Biosynthesis of other secondary metabolites	Phenylpropanoid biosynthesis	C05610, C00082, C00079, C00315, C05619, C02666	6	142	58	6490	0.1034	0.001567316	**	0.010003163	*	
ko00061	Metabolism	Lipid metabolism	Fatty acid biosynthesis	C00712, C00249, C01571, C00383, C01530, C02679	6	142	58	6490	0.1034	0.001567316	**	0.010003163	*	
ko01053	Metabolism	Metabolism of terpenoids and polyketides	Biosynthesis of siderophore group nonribosomal peptides	C00805, C00315, C00230, C00158	4	142	24	6490	0.1667	0.001660352	**	0.010294184	*	
ko01240	Metabolism	Global and overview maps	Biosynthesis of cofactors	C01825, C00072, C00864, C00255, C00064, C00082, C00153, C00078, C04294, C00026, C00254, C01040, C00025, C00378, C00049, C00158	16	142	328	6490	0.0488	0.002041516	**	0.012153183	*	
ko04977	Organismal Systems	Digestive system	Vitamin digestion and absorption	C00072, C00864, C00255, C00153, C00378	5	142	42	6490	0.119	0.002072202	**	0.012153183	*	
ko00592	Metabolism	Lipid metabolism	alpha-Linolenic acid metabolism	C16300, C08491, C16308, C16309, C00157	5	142	44	6490	0.1136	0.002554602	**	0.014588124	*	
ko00563	Metabolism	Glycan biosynthesis and metabolism	Glycosylphosphatidylinositol (GPI)-anchor biosynthesis	C00350, C01194	2	142	4	6490	0.5	0.002771158	**	0.015419005	*	
ko00240	Metabolism	Nucleotide metabolism	Pyrimidine metabolism	C02170, C00064, C00106, C00222, C00299, C00383	6	142	66	6490	0.0909	0.003050216	**	0.01654742	*	
ko00997	Metabolism	Biosynthesis of other secondary metabolites	Biosynthesis of various other secondary metabolites	C00082, C00079, C00078, C00026, C00025, C00049	6	142	67	6490	0.0896	0.003291073	**	0.017418607	*	
ko00562	Metabolism	Carbohydrate metabolism	Inositol phosphate metabolism	C03546, C00137, C00222, C01194, C00641	5	142	47	6490	0.1064	0.003424154	**	0.01769146	*	
ko04071	mental Information Pro	Signal transduction	Sphingolipid signaling pathway	C00212, C00195, C00836	3	142	15	6490	0.2	0.00384716	**	0.019414738	*	
ko00330	Metabolism	Amino acid metabolism	Arginine and proline metabolism	C01250, C00062, C15767, C00315, C01035, C00025	6	142	71	6490	0.0845	0.004398477	**	0.021692491	*	
ko04075	mental Information Pro	Signal transduction	Plant hormone signal transduction	C00805, C00954, C08491	3	142	16	6490	0.1875	0.004659778	**	0.022470484	*	
ko04964	Organismal Systems	Excretory system	Proximal tubule bicarbonate reclamation	C00064, C00026, C00025	3	142	17	6490	0.1765	0.005568544	**	0.026269	*	
ko00230	Metabolism	Nucleotide metabolism	Purine metabolism	C00212, C00242, C00387, C00064, C00147, C02353, C00559	7	142	101	6490	0.0693	0.006429628	**	0.029685731	*	
ko04140	Cellular Processes	Transport and catabolism	Autophagy - animal	C00350, C01194	2	142	6	6490	0.3333	0.006730847	**	0.029918765	*	
ko02020	mental Information Pro	Signal transduction	Two-component system	C00009, C00064, C00025, C00049, C00158	5	142	55	6490	0.0909	0.00675585	**	0.029918765	*	

ko04723	Organismal Systems	Nervous system	Retrograde endocannabinoid signaling	C00350, C00025, C00157	3	142	19	6490	0.1579	0.007685807	**	0.033356402	*	
ko00020	Metabolism	Carbohydrate metabolism	Citrate cycle (TCA cycle)	C00417, C00026, C00158	3	142	20	6490	0.15	0.00889914	**	0.037864967	*	
ko00480	Metabolism	Metabolism of other amino acids	Glutathione metabolism	C00072, C01879, C00315, C00025	4	142	38	6490	0.1053	0.009106771	**	0.038003256	*	
ko05030	Human Diseases	Substance dependence	Cocaine addiction	C00082, C00025	2	142	7	6490	0.2857	0.00928856	**	0.038030519	*	
ko00946	Metabolism	Biosynthesis of other secondary metabolites	Degradation of flavonoids	C01617, C00774, C02183, C00389	4	142	40	6490	0.1	0.010902911	*	0.043660397	*	
ko00627	Metabolism	Xenobiotics biodegradation and metabolism	Aminobenzoate degradation	C06337, C00146, C02183, C00230, C00472, C00090	6	142	86	6490	0.0698	0.011065999	*	0.043660397	*	
ko04724	Organismal Systems	Nervous system	Glutamatergic synapse	C00064, C00025	2	142	8	6490	0.25	0.012208119	*	0.047306463	*	
ko00630	Metabolism	Carbohydrate metabolism	Glyoxylate and dicarboxylate metabolism	C00417, C00064, C00026, C00025, C00158	5	142	64	6490	0.0781	0.012668101	*	0.048021084	*	
ko01063	Metabolism	Chemical structure transformation maps	Biosynthesis of alkaloids derived from shikimate pathway	C00417, C00082, C00079, C00078, C00026, C16700, C00254, C00158	8	142	143	6490	0.0559	0.012835128	*	0.048021084	*	
ko00720	Metabolism	Energy metabolism	Other carbon fixation pathways	C00417, C00026, C00222, C00158	4	142	44	6490	0.0909	0.015146031	*	0.055042204	ns	
ko05031	Human Diseases	Substance dependence	Amphetamine addiction	C00082, C00025	2	142	9	6490	0.2222	0.015472693	*	0.055042204	ns	
ko04730	Organismal Systems	Nervous system	Long-term depression	C00025, C00641	2	142	9	6490	0.2222	0.015472693	*	0.055042204	ns	
ko00999	Metabolism	Biosynthesis of other secondary metabolites	Biosynthesis of various plant secondary metabolites	C10682, C00082, C00079, C05839, C00078, C00254, C09265, C00049	8	142	149	6490	0.0537	0.016125786	*	0.056227582	ns	
ko00460	Metabolism	Metabolism of other amino acids	Cyanoamino acid metabolism	C00082, C00079, C00152, C00049	4	142	45	6490	0.0889	0.016348164	*	0.056227582	ns	
ko04024	mental Information Pr	Signal transduction	cAMP signaling pathway	C00212, C00416, C20793	3	142	25	6490	0.12	0.01658325	*	0.056227582	ns	
ko00052	Metabolism	Carbohydrate metabolism	Galactose metabolism	C00984, C00137, C00089, C00492	4	142	46	6490	0.087	0.017608585	*	0.058785583	ns	
ko05012	Human Diseases	Neurodegenerative disease	Parkinson disease	C00212, C00009, C00082	3	142	26	6490	0.1154	0.018451742	*	0.06066709	ns	
ko00650	Metabolism	Carbohydrate metabolism	Butanoate metabolism	C00497, C00026, C00025, C01384	4	142	47	6490	0.0851	0.018928067	*	0.060842983	ns	
ko04212	Organismal Systems	Aging	Longevity regulating pathway - worm	C00712, C00153	2	142	10	6490	0.2	0.019066004	*	0.060842983	ns	
ko00960	Metabolism	Biosynthesis of other secondary metabolites	Tropane, piperidine and pyridine alkaloid biosynthesis	C00408, C00079, C05607, C01746, C01479	5	142	72	6490	0.0694	0.020249349	*	0.06334048	ns	
ko00073	Metabolism	Lipid metabolism	Cutin, suberine and wax biosynthesis	C00712, C00249, C19615	3	142	27	6490	0.1111	0.020432413	*	0.06334048	ns	
ko00360	Metabolism	Amino acid metabolism	Phenylalanine metabolism	C03519, C00082, C00079, C05607	4	142	49	6490	0.0816	0.021747018	*	0.066466238	ns	
ko00941	Metabolism	Biosynthesis of other secondary metabolites	Flavonoid biosynthesis	C01617, C00774, C09727, C09320, C00389	5	142	74	6490	0.0676	0.022524784	*	0.067887196	ns	
ko04072	mental Information Pr	Signal transduction	Phospholipase D signaling pathway	C00416, C00025	2	142	11	6490	0.1818	0.022972312	*	0.068287557	ns	
ko00770	Metabolism	Metabolism of cofactors and vitamins	Pantothenate and CoA biosynthesis	C00864, C00106, C00049	3	142	30	6490	0.1	0.027049228	*	0.078630387	ns	
ko04721	Organismal Systems	Nervous system	Synaptic vesicle cycle	C00025, C00388	2	142	12	6490	0.1667	0.027176401	*	0.078630387	ns	
ko00350	Metabolism	Amino acid metabolism	Tyrosine metabolism	C00082, C00146, C05581, C05580, C01384	5	142	78	6490	0.0641	0.027559161	*	0.078688656	ns	
ko04080	mental Information Pr	Signaling molecules and interaction	Neuroactive ligand-receptor interaction	C00212, C00025, C00388, C00049	4	142	53	6490	0.0755	0.028121309	*	0.079250961	ns	
ko00730	Metabolism	Metabolism of cofactors and vitamins	Thiamine metabolism	C00082, C04294, C00378	3	142	31	6490	0.0968	0.029479418	*	0.082013253	ns	

ko00998	Metabolism	Biosynthesis of other secondary metabolites	Biosynthesis of various antibiotics	C00062, C00082, C00026, C00254, C00025, C00049	6	142	108	6490	0.0556	0.030576037	*	0.083987343	ns	
ko00760	Metabolism	Metabolism of cofactors and vitamins	Nicotinate and nicotinamide metabolism	C19569, C00153, C00049, C01384	4	142	55	6490	0.0727	0.031684023	*	0.084739301	ns	
ko05022	Human Diseases	Neurodegenerative disease	Pathways of neurodegeneration - multiple diseases	C00009, C00062, C00025	3	142	32	6490	0.0938	0.032021303	*	0.084739301	ns	
ko04742	Organismal Systems	Sensory system	Taste transduction	C00089, C00025, C00158	3	142	32	6490	0.0938	0.032021303	*	0.084739301	ns	
ko01200	Metabolism	Global and overview maps	Carbon metabolism	C00257, C00026, C00222, C00025, C00049, C00158	6	142	112	6490	0.0536	0.03564231	*	0.093185316	ns	
ko05014	Human Diseases	Neurodegenerative disease	Amyotrophic lateral sclerosis	C00062, C00025	2	142	14	6490	0.1429	0.036419584	*	0.094083925	ns	
ko00600	Metabolism	Lipid metabolism	Sphingolipid metabolism	C00195, C00836, C01190	3	142	35	6490	0.0857	0.040310483	*	0.10171366	ns	
ko00660	Metabolism	Carbohydrate metabolism	C5-Branched dibasic acid metabolism	C00417, C00026, C00025	3	142	35	6490	0.0857	0.040310483	*	0.10171366	ns	
ko04066	mental Information Pro	Signal transduction	HIF-1 signaling pathway	C00072, C00026	2	142	15	6490	0.1333	0.041430726	*	0.103338708	ns	
ko04111	Cellular Processes	Cell growth and death	Cell cycle - yeast	C00009	1	142	2	6490	0.5	0.043284202	*	0.10436302	ns	
ko05212	Human Diseases	Cancer: specific types	Pancreatic cancer	C00416	1	142	2	6490	0.5	0.043284202	*	0.10436302	ns	
ko00571	Metabolism	Glycan biosynthesis and metabolism	Lipoarabinomannan (LAM) biosynthesis	C01194	1	142	2	6490	0.5	0.043284202	*	0.10436302	ns	
ko00261	Metabolism	Biosynthesis of other secondary metabolites	Monobactam biosynthesis	C00062, C00082, C00049	3	142	39	6490	0.0769	0.052875191	ns	0.126086994	ns	
ko00640	Metabolism	Carbohydrate metabolism	Propanoate metabolism	C04593, C02170, C00222	3	142	41	6490	0.0732	0.059783421	ns	0.141010896	ns	
ko04931	Human Diseases	Endocrine and metabolic disease	Insulin resistance	C00195, C02571	2	142	19	6490	0.1053	0.06376778	ns	0.148260447	ns	
ko05130	Human Diseases	Infectious disease: bacterial	Pathogenic Escherichia coli infection	C00350	1	142	3	6490	0.3333	0.064223419	ns	0.148260447	ns	
ko00910	Metabolism	Energy metabolism	Nitrogen metabolism	C00064, C00025	2	142	20	6490	0.1	0.069864296	ns	0.15958476	ns	
ko04148	Cellular Processes	Transport and catabolism	Efferocytosis	C00062, C04230	2	142	21	6490	0.0952	0.076142819	ns	0.172114498	ns	
ko01040	Metabolism	Lipid metabolism	Biosynthesis of unsaturated fatty acids	C00712, C00249, C01530, C01595	4	142	74	6490	0.0541	0.078200332	ns	0.174943011	ns	
ko04150	mental Information Pro	Signal transduction	mTOR signaling pathway	C00062	1	142	4	6490	0.25	0.084707507	ns	0.187566622	ns	
ko01066	Metabolism	Chemical structure transformation maps	Biosynthesis of alkaloids derived from terpenoid and polyketide	C00417, C00026, C00158	3	142	48	6490	0.0625	0.087030351	ns	0.188176791	ns	
ko00260	Metabolism	Amino acid metabolism	Glycine, serine and threonine metabolism	C00078, C00114, C00049	3	142	48	6490	0.0625	0.087030351	ns	0.188176791	ns	
ko00362	Metabolism	Xenobiotics biodegradation and metabolism	Benzoate degradation	C00146, C00230, C02480, C00090	4	142	77	6490	0.0519	0.087584589	ns	0.188176791	ns	
ko00290	Metabolism	Amino acid metabolism	Valine, leucine and isoleucine biosynthesis	C02631, C02504	2	142	23	6490	0.087	0.089203254	ns	0.18977555	ns	
ko00996	Metabolism	Biosynthesis of other secondary metabolites	Biosynthesis of various alkaloids	C00805, C00079, C00078, C00254	4	142	78	6490	0.0513	0.090829207	ns	0.191358621	ns	
ko00430	Metabolism	Metabolism of other amino acids	Taurine and hypotaurine metabolism	C00026, C00025	2	142	24	6490	0.0833	0.09596478	ns	0.200234204	ns	
ko00565	Metabolism	Lipid metabolism	Ether lipid metabolism	C00670, C00958	2	142	25	6490	0.08	0.102867522	ns	0.204774274	ns	
ko04722	Organismal Systems	Nervous system	Neurotrophin signaling pathway	C00195	1	142	5	6490	0.2	0.104746288	ns	0.204774274	ns	

ko05167	Human Diseases	Infectious disease: viral	Kaposi sarcoma-associated herpesvirus infection	C00350	1	142	5	6490	0.2	0.104746288	ns	0.204774274	ns	
ko03320	Organismal Systems	Endocrine system	PPAR signaling pathway	C14767	1	142	5	6490	0.2	0.104746288	ns	0.204774274	ns	
ko04068	mental Information Pro	Signal transduction	FoxO signaling pathway	C00025	1	142	5	6490	0.2	0.104746288	ns	0.204774274	ns	
ko05310	Human Diseases	Immune disease	Asthma	C00388	1	142	5	6490	0.2	0.104746288	ns	0.204774274	ns	
ko05132	Human Diseases	Infectious disease: bacterial	Salmonella infection	C01194	1	142	5	6490	0.2	0.104746288	ns	0.204774274	ns	
ko04922	Organismal Systems	Endocrine system	Glucagon signaling pathway	C00026, C00158	2	142	26	6490	0.0769	0.10990217	ns	0.212935455	ns	
ko04973	Organismal Systems	Digestive system	Carbohydrate digestion and absorption	C01835, C00089	2	142	27	6490	0.0741	0.117059751	ns	0.224796158	ns	
ko05140	Human Diseases	Infectious disease: parasitic	Leishmaniasis	C00195	1	142	6	6490	0.1667	0.124349376	ns	0.226754745	ns	
ko04912	Organismal Systems	Endocrine system	GnRH signaling pathway	C00416	1	142	6	6490	0.1667	0.124349376	ns	0.226754745	ns	
ko05142	Human Diseases	Infectious disease: parasitic	Chagas disease	C00062	1	142	6	6490	0.1667	0.124349376	ns	0.226754745	ns	
ko04916	Organismal Systems	Endocrine system	Melanogenesis	C00082	1	142	6	6490	0.1667	0.124349376	ns	0.226754745	ns	
ko05016	Human Diseases	Neurodegenerative disease	Huntington disease	C00025	1	142	6	6490	0.1667	0.124349376	ns	0.226754745	ns	
ko02030	Cellular Processes	Cell motility	Bacterial chemotaxis	C00049	1	142	6	6490	0.1667	0.124349376	ns	0.226754745	ns	
ko02060	mental Information Pro	Membrane transport	Phosphotransferase system (PTS)	C00072, C00089, C01083	3	142	57	6490	0.0526	0.128245481	ns	0.231910577	ns	
ko04361	Organismal Systems	Development and regeneration	Axon regeneration	C00078	1	142	7	6490	0.1429	0.143526179	ns	0.251170813	ns	
ko04720	Organismal Systems	Nervous system	Long-term potentiation	C00025	1	142	7	6490	0.1429	0.143526179	ns	0.251170813	ns	
ko05017	Human Diseases	Neurodegenerative disease	Spinocerebellar ataxia	C00025	1	142	7	6490	0.1429	0.143526179	ns	0.251170813	ns	
ko05033	Human Diseases	Substance dependence	Nicotine addiction	C00025	1	142	7	6490	0.1429	0.143526179	ns	0.251170813	ns	
ko00620	Metabolism	Carbohydrate metabolism	Pyruvate metabolism	C02488, C02504	2	142	32	6490	0.0625	0.154399716	ns	0.265910622	ns	
ko00401	Metabolism	Biosynthesis of other secondary metabolites	Novobiocin biosynthesis	C00082, C00254	2	142	32	6490	0.0625	0.154399716	ns	0.265910622	ns	
ko05032	Human Diseases	Substance dependence	Morphine addiction	C00212	1	142	8	6490	0.125	0.162285902	ns	0.269055371	ns	
ko04920	Organismal Systems	Endocrine system	Adipocytokine signaling pathway	C00195	1	142	8	6490	0.125	0.162285902	ns	0.269055371	ns	
ko04666	Organismal Systems	Immune system	Fc gamma R-mediated phagocytosis	C00416	1	142	8	6490	0.125	0.162285902	ns	0.269055371	ns	
ko05143	Human Diseases	Infectious disease: parasitic	African trypanosomiasis	C00078	1	142	8	6490	0.125	0.162285902	ns	0.269055371	ns	
ko04976	Organismal Systems	Digestive system	Bile secretion	C00805, C00114, C00026, C00315	4	142	97	6490	0.0412	0.162425132	ns	0.269055371	ns	
ko00300	Metabolism	Amino acid metabolism	Lysine biosynthesis	C00026, C00049	2	142	35	6490	0.0571	0.177771761	ns	0.29035814	ns	
ko04933	Human Diseases	Endocrine and metabolic disease	AGE-RAGE signaling pathway in diabetic complications	C00195	1	142	9	6490	0.1111	0.180637553	ns	0.29035814	ns	
ko04713	Organismal Systems	Environmental adaptation	Circadian entrainment	C00025	1	142	9	6490	0.1111	0.180637553	ns	0.29035814	ns	
ko05152	Human Diseases	Infectious disease: bacterial	Tuberculosis	C01194	1	142	9	6490	0.1111	0.180637553	ns	0.29035814	ns	
ko00500	Metabolism	Carbohydrate metabolism	Starch and sucrose metabolism	C00089, C01083	2	142	37	6490	0.0541	0.193642448	ns	0.305631337	ns	
ko04022	mental Information Pro	Signal transduction	cGMP-PKG signaling pathway	C00212	1	142	10	6490	0.1	0.198589947	ns	0.305631337	ns	
ko04217	Cellular Processes	Cell growth and death	Necroptosis	C00195	1	142	10	6490	0.1	0.198589947	ns	0.305631337	ns	
ko00331	Metabolism	Biosynthesis of other secondary metabolites	Clavulanic acid biosynthesis	C00062	1	142	10	6490	0.1	0.198589947	ns	0.305631337	ns	
ko07227	Drug Development	Target-based classification: G protein-coupled receptors	Histamine H2/H3 receptor agonists/antagonists	C00388	1	142	10	6490	0.1	0.198589947	ns	0.305631337	ns	
ko04261	Organismal Systems	Circulatory system	Adrenergic signaling in cardiomyocytes	C07056	1	142	10	6490	0.1	0.198589947	ns	0.305631337	ns	

ko00624	Metabolism	Xenobiotics biodegradation and metabolism	Polycyclic aromatic hydrocarbon degradation	C00805, C06337, C00230, C00090	4	142	107	6490	0.0374	0.206369123	ns	0.312699474	ns	
ko00908	Metabolism	Metabolism of terpenoids and polyketides	Zeatin biosynthesis	C00147, C00170	2	142	39	6490	0.0513	0.209687844	ns	0.312699474	ns	
ko00195	Metabolism	Energy metabolism	Photosynthesis	C00009	1	142	11	6490	0.0909	0.21615171	ns	0.312699474	ns	
ko04928	Organismal Systems	Endocrine system	Parathyroid hormone synthesis, secretion and action	C00009	1	142	11	6490	0.0909	0.21615171	ns	0.312699474	ns	
ko04917	Organismal Systems	Endocrine system	Prolactin signaling pathway	C00082	1	142	11	6490	0.0909	0.21615171	ns	0.312699474	ns	
ko04540	Cellular Processes	Cellular community - eukaryotes	Gap junction	C00025	1	142	11	6490	0.0909	0.21615171	ns	0.312699474	ns	
ko04664	Organismal Systems	Immune system	Fc epsilon RI signaling pathway	C00388	1	142	11	6490	0.0909	0.21615171	ns	0.312699474	ns	
ko04122	etic Information Proces	Folding, sorting and degradation	Sulfur relay system	C00378	1	142	11	6490	0.0909	0.21615171	ns	0.312699474	ns	
ko04745	Organismal Systems	Sensory system	Phototransduction - fly	C01530	1	142	11	6490	0.0909	0.21615171	ns	0.312699474	ns	
ko00190	Metabolism	Energy metabolism	Oxidative phosphorylation	C00009	1	142	12	6490	0.0833	0.233331284	ns	0.330933913	ns	
ko04728	Organismal Systems	Nervous system	Dopaminergic synapse	C00082	1	142	12	6490	0.0833	0.233331284	ns	0.330933913	ns	
ko04725	Organismal Systems	Nervous system	Cholinergic synapse	C00114	1	142	12	6490	0.0833	0.233331284	ns	0.330933913	ns	
ko00966	Metabolism	Biosynthesis of other secondary metabolites	Glucosinolate biosynthesis	C00082, C00079, C00078	3	142	77	6490	0.039	0.237280515	ns	0.333027659	ns	
ko01110	Metabolism	Global and overview maps	Biosynthesis of secondary metabolites	C01250, C18021, C16300, C01825, C00408, C00072, C05610, C00416, C01617, C00805, C00774, C00417, C11918, C00864, C10682, C00255, C02631, C00062, C08491, C00064, C00082, C00350, C16308, C00135, C00079, C05839, C09727, C02504, C00147, C00078, C00152, C00257, C16309, C05607, C05437, C00026, C00137, C09320, C00254, C09265, C00315, C00089, C00230, C00025, C00388, C00049, C05619, C02666, C00158, C00641, C00389, C01083, C00157, C01479, C02679	55	142	2308	6490	0.0238	0.237876899	ns	0.333027659	ns	
ko00625	Metabolism	Xenobiotics biodegradation and metabolism	Chloroalkane and chloroalkene degradation	C06752, C00222	2	142	43	6490	0.0465	0.242128416	ns	0.336057765	ns	
ko05204	Human Diseases	Cancer: overview	Chemical carcinogenesis - DNA adducts	C06752, C19569, C19603	3	142	78	6490	0.0385	0.243138568	ns	0.336057765	ns	
ko04975	Organismal Systems	Digestive system	Fat digestion and absorption	C00416	1	142	13	6490	0.0769	0.250136927	ns	0.341381844	ns	
ko05146	Human Diseases	Infectious disease: parasitic	Amoebiasis	C00062	1	142	13	6490	0.0769	0.250136927	ns	0.341381844	ns	
ko04923	Organismal Systems	Endocrine system	Regulation of lipolysis in adipocytes	C00212	1	142	14	6490	0.0714	0.266576723	ns	0.359299061	ns	
ko04971	Organismal Systems	Digestive system	Gastric acid secretion	C00388	1	142	14	6490	0.0714	0.266576723	ns	0.359299061	ns	
ko00380	Metabolism	Amino acid metabolism	Tryptophan metabolism	C02693, C00954, C00078	3	142	83	6490	0.0361	0.272748841	ns	0.365348756	ns	
ko00404	Metabolism	Biosynthesis of other secondary metabolites	Staurosporine biosynthesis	C00135, C00078	2	142	48	6490	0.0417	0.282941172	ns	0.376676285	ns	

ko04270	Organismal Systems	Circulatory system	Vascular smooth muscle contraction	C00212	1	142	16	6490	0.0625	0.298390237	ns	0.394821229	ns	
ko00944	Metabolism	Biosynthesis of other secondary metabolites	Flavone and flavonol biosynthesis	C01750, C00389	2	142	51	6490	0.0392	0.307370981	ns	0.404239412	ns	
ko04924	Organismal Systems	Endocrine system	Renin secretion	C00212	1	142	17	6490	0.0588	0.313779268	ns	0.410181333	ns	
ko00333	Metabolism	Biosynthesis of other secondary metabolites	Prodigiosin biosynthesis	C02679	1	142	18	6490	0.0556	0.328833083	ns	0.427286102	ns	
ko01055	Metabolism	Metabolism of terpenoids and polyketides	Biosynthesis of vancomycin group antibiotics	C00082	1	142	19	6490	0.0526	0.343558933	ns	0.443763622	ns	
ko00310	Metabolism	Amino acid metabolism	Lysine degradation	C00408, C00026	2	142	56	6490	0.0357	0.34769528	ns	0.446448969	ns	
ko01220	Metabolism	Global and overview maps	Degradation of aromatic compounds	C07215, C00805, C06337, C00146, C00230, C02480, C00090	7	142	263	6490	0.0266	0.352945195	ns	0.45052416	ns	
ko05208	Human Diseases	Cancer: overview	Chemical carcinogenesis - reactive oxygen species	C00146, C00472	2	142	57	6490	0.0351	0.355675273	ns	0.451354001	ns	
ko01062	Metabolism	Chemical structure transformation maps	Biosynthesis of terpenoids and steroids	C00417, C00026, C00158	3	142	98	6490	0.0306	0.362966568	ns	0.457928752	ns	
ko00710	Metabolism	Energy metabolism	Carbon fixation by Calvin cycle	C00049	1	142	21	6490	0.0476	0.372054969	ns	0.466681666	ns	
ko00626	Metabolism	Xenobiotics biodegradation and metabolism	Naphthalene degradation	C00805, C00090	2	142	60	6490	0.0333	0.379400497	ns	0.47316039	ns	
ko04152	mental Information Pro	Signal transduction	AMPK signaling pathway	C00389	1	142	22	6490	0.0455	0.385838891	ns	0.478440225	ns	
ko04714	Organismal Systems	Environmental adaptation	Thermogenesis	C02301	1	142	23	6490	0.0435	0.399322329	ns	0.489564664	ns	
ko00965	Metabolism	Biosynthesis of other secondary metabolites	Betalain biosynthesis	C00082	1	142	23	6490	0.0435	0.399322329	ns	0.489564664	ns	
ko01120	Metabolism	Global and overview maps	Microbial metabolism in diverse environments	C07215, C06752, C00408, C00072, C00805, C00417, C06337, C19569, C00064, C00146, C00257, C00026, C00137, C02183, C00222, C00230, C00025, C02480, C00049, C11101, C00158, C00472, C00090, C01546, C01384	25	142	1075	6490	0.0233	0.4026464	ns	0.490866679	ns	
ko00740	Metabolism	Metabolism of cofactors and vitamins	Riboflavin metabolism	C00255	1	142	24	6490	0.0417	0.412511788	ns	0.497305878	ns	
ko00521	Metabolism	Biosynthesis of other secondary metabolites	Streptomycin biosynthesis	C00137	1	142	24	6490	0.0417	0.412511788	ns	0.497305878	ns	
ko00621	Metabolism	Xenobiotics biodegradation and metabolism	Dioxin degradation	C00805, C00090	2	142	66	6490	0.0303	0.425698745	ns	0.510368108	ns	
ko00270	Metabolism	Amino acid metabolism	Cysteine and methionine metabolism	C00170, C00049	2	142	68	6490	0.0294	0.440738654	ns	0.525496087	ns	
ko00750	Metabolism	Metabolism of cofactors and vitamins	Vitamin B6 metabolism	C00064	1	142	29	6490	0.0345	0.474267442	ns	0.559326277	ns	
ko04216	Cellular Processes	Cell growth and death	Ferroptosis	C00025	1	142	29	6490	0.0345	0.474267442	ns	0.559326277	ns	
ko05200	Human Diseases	Cancer: overview	Pathways in cancer	C00416	1	142	31	6490	0.0323	0.497124368	ns	0.583113448	ns	
ko00332	Metabolism	Biosynthesis of other secondary metabolites	Carbapenem biosynthesis	C00025	1	142	32	6490	0.0312	0.508180002	ns	0.589706206	ns	
ko00364	Metabolism	Xenobiotics biodegradation and metabolism	Fluorobenzoate degradation	C00090	1	142	32	6490	0.0312	0.508180002	ns	0.589706206	ns	
ko00074	Metabolism	Lipid metabolism	Mycolic acid biosynthesis	C01083	1	142	34	6490	0.0294	0.529572358	ns	0.611261711	ns	

ko04750	Organismal Systems	Sensory system	Inflammatory mediator regulation of TRP channels	C00388	1	142	35	6490	0.0286	0.539919435	ns	0.619907499	ns	
ko00361	Metabolism	Xenobiotics biodegradation and metabolism	Chlorocyclohexane and chlorobenzene degradation	C00146, C00090	2	142	83	6490	0.0241	0.54610929	ns	0.623714295	ns	
ko00030	Metabolism	Carbohydrate metabolism	Pentose phosphate pathway	C00257	1	142	37	6490	0.027	0.55994044	ns	0.636162699	ns	
ko00622	Metabolism	Xenobiotics biodegradation and metabolism	Xylene degradation	C07215	1	142	38	6490	0.0263	0.569624069	ns	0.64379387	ns	
ko05415	Human Diseases	Cardiovascular disease	Diabetic cardiomyopathy	C00195	1	142	39	6490	0.0256	0.579096075	ns	0.651108022	ns	
ko00062	Metabolism	Lipid metabolism	Fatty acid elongation	C00249	1	142	40	6490	0.025	0.58836105	ns	0.658115196	ns	
ko00280	Metabolism	Amino acid metabolism	Valine, leucine and isoleucine degradation	C02170	1	142	42	6490	0.0238	0.606287788	ns	0.671247194	ns	
ko04726	Organismal Systems	Nervous system	Serotonergic synapse	C00078	1	142	42	6490	0.0238	0.606287788	ns	0.671247194	ns	
ko00785	Metabolism	Metabolism of cofactors and vitamins	Lipoic acid metabolism	C00026	1	142	44	6490	0.0227	0.623439082	ns	0.68673239	ns	
ko00860	Metabolism	Metabolism of cofactors and vitamins	Porphyrin metabolism	C18021, C00025, C02800	3	142	148	6490	0.0203	0.633748777	ns	0.694563054	ns	
ko00623	Metabolism	Xenobiotics biodegradation and metabolism	Toluene degradation	C00230	1	142	47	6490	0.0213	0.647784552	ns	0.70637813	ns	
ko00071	Metabolism	Lipid metabolism	Fatty acid degradation	C00249	1	142	50	6490	0.02	0.670566405	ns	0.727564549	ns	
ko02024	Cellular Processes	Cellular community - prokaryotes	Quorum sensing	C00009	1	142	55	6490	0.0182	0.705329528	ns	0.761475162	ns	
ko00100	Metabolism	Lipid metabolism	Steroid biosynthesis	C05437	1	142	57	6490	0.0175	0.71819191	ns	0.771522992	ns	
ko00523	Metabolism	Metabolism of terpenoids and polyketides	Polyketide sugar unit biosynthesis	C11918	1	142	58	6490	0.0172	0.724412452	ns	0.774371932	ns	
ko00040	Metabolism	Carbohydrate metabolism	Pentose and glucuronate interconversions	C00026	1	142	59	6490	0.0169	0.73049663	ns	0.777047886	ns	
ko00980	Metabolism	Xenobiotics biodegradation and metabolism	Metabolism of xenobiotics by cytochrome P450	C19569, C19603	2	142	121	6490	0.0165	0.747938702	ns	0.789930438	ns	
ko01212	Metabolism	Global and overview maps	Fatty acid metabolism	C00249, C00383	2	142	122	6490	0.0164	0.752034385	ns	0.789930438	ns	
ko01059	Metabolism	Metabolism of terpenoids and polyketides	Biosynthesis of enediyne antibiotics	C00082	1	142	63	6490	0.0159	0.753528114	ns	0.789930438	ns	
ko00950	Metabolism	Biosynthesis of other secondary metabolites	Isoquinoline alkaloid biosynthesis	C00082, C16700	2	142	131	6490	0.0153	0.786395429	ns	0.820422154	ns	
ko00130	Metabolism	Metabolism of cofactors and vitamins	Ubiquinone and other terpenoid-quinone biosynthesis	C00082	1	142	71	6490	0.0141	0.793889335	ns	0.824277444	ns	
ko00590	Metabolism	Lipid metabolism	Arachidonic acid metabolism	C00157	1	142	79	6490	0.0127	0.827680035	ns	0.85526937	ns	
ko00524	Metabolism	Biosynthesis of other secondary metabolites	Neomycin, kanamycin and gentamicin biosynthesis	C00025	1	142	81	6490	0.0123	0.835229656	ns	0.858980262	ns	
ko00982	Metabolism	Xenobiotics biodegradation and metabolism	Drug metabolism - cytochrome P450	C16651	1	142	87	6490	0.0115	0.855963506	ns	0.875292237	ns	
ko00680	Metabolism	Energy metabolism	Methane metabolism	C00082	1	142	88	6490	0.0114	0.859157818	ns	0.875292237	ns	
ko00901	Metabolism	Biosynthesis of other secondary metabolites	Indole alkaloid biosynthesis	C00078	1	142	104	6490	0.0096	0.901669918	ns	0.914310151	ns	
ko00520	Metabolism	Carbohydrate metabolism	Amino sugar and nucleotide sugar metabolism	C00984	1	142	119	6490	0.0084	0.929849065	ns	0.937244034	ns	

ko00906	Metabolism	Metabolism of terpenoids and polyketides	Carotenoid biosynthesis	C15971	1	142	123	6490	0.0081	0.935898671	ns	0.937244034	ns	
ko01250	Metabolism	Global and overview maps	Biosynthesis of nucleotide sugars	C00984, C00137	2	142	200	6490	0.01	0.937244034	ns	0.937244034	ns	

Note:Significance levels are indicated by ****, ***, **, and * for p–value ranges of [0.00001, 0.0001),[0.0001, 0.001), [0.001, 0.01), and [0.01, 0.05), respectively.
