# Supplementary material for: Integrated Transcriptomic and Metabolomic Analyses Reveal the Regulatory Networks of Cuticular Wax Fatty Acid Metabolism in Jujube (Ziziphus jujuba Mill. ‘Hupingzao’)
Source: Life (Basel). 2026 Oct 9;16(10):1685. doi: 10.3390/life16101685 (PMC13706060; doi:10.3390/life16101685)
Supplement: Supplementary file 1 [file life-16-01685-s001.zip › Table S5.pdf]

Table S5 The proportional composition of wax components in 'Hupingzao' jujube fruits.

	WR	FR
Alkanes	7.735384909	1.201130993
Aldehydes	3.624718623	12.29092005
Fatty acids	57.81933965	30.27399281
Esters	0.473116151	21.80237119
Ketones	0	6.383018785
Phenols	0.471283078	0.168048503
Primary alcohols	27.72926244	26.70276597
Amines	2.146895141	1.177751696

Note:The wax components were considered as a whole, and only their relative proportions are presented. The percentages were normalized to 100% after removing impurities from the GC-MS data.
